# Lewis‐Acid Engineering with Neodymium Promoters as Synergistic Nd‐Ni Dual Sites for Enhanced Urea Oxidation

**DOI:** 10.1002/advs.75788

**Published:** 2026-05-22

**Authors:** Mingfan Li, Jun Mei, Jiahao Zhao, Ruipeng Guo, Jing Shang, Ziyou Dong, Juan Bai, Qianqian Yao, Shixue Dou

**Affiliations:** ^1^ National & Local Joint Engineering Research Center For High‐efficiency Display and Lighting Technology, Key Laboratory for Special Functional Materials of Ministry of Education, School of Nanoscience and Materials Engineering Henan University Zhengzhou China; ^2^ School of Materials Science and Engineering Shaanxi University of Science and Technology Xi'an China; ^3^ School of Chemistry and Physics Queensland University of Technology Brisbane QLD Australia; ^4^ Institute of Energy Materials Science University of Shanghai for Science and Technology Shanghai China; ^5^ Institute for Superconducting and Electronic Materials University of Wollongong Wollongong New South Wales Australia

**Keywords:** electrocatalyst, Lewis acid, neodymium, nickel, urea

## Abstract

The sluggish kinetics of the urea oxidation reaction (UOR) are strongly associated with the high energy barriers required for the C‐N cleavage and N‐N coupling steps. While nickel‐based catalysts are promising, their performance is limited by high operational potentials and structural instability. This work introduces neodymium (Nd) as a Lewis acid site promoter into a nickel‐based framework to create Nd‐Ni oxide catalysts. The strong Lewis acidity of Nd centers enhances urea adsorption and facilitates C‐N cleavage and N‐N coupling, while the significant electronegativity difference between Nd and Ni induces electronic redistribution for optimizing intermediate adsorption for UOR. As a result, the optimal catalyst demonstrates superior UOR performance, in which a low potential of 1.347 V is required to achieve 10 mA cm^−2^ accompanied by excellent cycling stability. When integrated into a Zn‐urea battery, it delivers a peak power density of 11.6 mW cm^−2^. This study highlights the critical role of Lewis‐acid engineering via rare‐earth metal incorporation in restructuring reaction pathways, and offers a promising strategy for advancing renewable energy conversion.

## Graphical abstract

The integration of rare‐metal neodymium centers to engineer Lewis acid sites with nickel centers is proposed as highly efficient UOR catalysts.

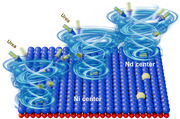

## 1 Introduction

The accumulation of greenhouse gases for driving future climate change and an ever‐increasing demand for clean energy are two dominate crises, which requires a fundamental transition from a fossil‐fuel‐based economy to a sustainable one powered by renewable resources [1]. Hydrogen, which can be obtained through carbon‐neutral water electrolysis rationally coupled with renewable electricity sources such as solar or wind, has emerged as a potential energy carrier [2]. Generally, the two‐electron hydrogen evolution reaction (HER) at cathode is relatively efficient, however, the four‐electron oxygen evolution reaction (OER) at the anodic counterpart is a bottleneck, resulting in high overpotentials [3]. Hence, replacing OER with some energy‐saving reactions, such as urea oxidation reaction (UOR) is one of the most promising solutions [4, 5]. Specifically, the thermodynamic potential for UOR (0.37 V vs. RHE) is significantly lower than that of OER (1.23 V vs. RHE), suggesting that a much lower voltage is required to produce hydrogen from urea‐rich water, and meanwhile this reaction process can effectively purify wastewater through oxidizing the toxic urea molecule. However, the practical application of UOR is complex. First, its mechanism involves a six‐electron transfer process with multiple adsorbed intermediates (e.g. *CO, *NH, and *CN), leading to sluggish kinetics compared to OER. Also, the cleavage of the strong C‐N bond (≈293 kJ mol^−1^) in the urea molecule is difficult [6]. Consequently, the development of highly active and stable catalysts is beneficial to unlock the full potential of UOR‐involved hydrogen production and environmental remediation [7].

Currently, efficient UOR electrocatalysts are primarily categorized into noble metal‐based and non‐noble metal‐based catalysts. Noble metals, such as Rh and Pt, demonstrate attractive intrinsic activity for UOR, but the extreme natural scarcity and high manufacture costs greatly limit their large‐scale utilization. Non‐noble metal‐based catalysts, particularly earth‐abundant transition metals, offer a compelling compromise between production costs and catalytic activities [8, 9, 10]. For example, nickel (Ni) has been evidenced as a cost‐effective candidate, which is largely dependent on the Ni^2+^/Ni^3+^ redox couples [11], in which Ni^3+^ sites act as strong Lewis acids that adsorb and activate urea molecules. To date, various Ni‐based catalysts, including different dimensional nanostructures, compositional alloys, and types of hybrids, have been rationally synthesized for optimizing UOR performance [12, 13].

Unfortunately, several major challenges remain, such as the high operational potentials for achieving appreciable current densities, the limited densities for the rate‐determining steps, the undesired intermediate adsorption for catalyst deactivation, and the reversible structural degradation for performance decay [14]. Therefore, some modulation strategies, such as vacancy and defect engineering, are essential for further performance enhancement. On the other hand, from the mechanism insight, it is well recognized that the presence of Lewis acid sites is essential for an efficient UOR catalyst, which favors the effective activating urea molecules and enabling the key dehydrogenation steps by interacting with hydroxide ions in alkaline solutions [15]. However, studies on Lewis acid engineering are quite limited for UOR. Thus, to boost activity by adjusting Lewis acid sites is a potential solution for UOR catalyst design.

In this work, a secondary rare‐metal center, neodymium (Nd), that can function as a Lewis acid site promoter is introduced into Ni‐based catalysts. As a typical rare earth element, owing to the high charge density and large ionic radius, Nd centers manifest strong Lewis acidity, which is expected to strongly interact with Lewis basic sites of urea molecules (‐NH_2_), thus facilitating urea adsorption and promoting N‐H cleavage [16, 17, 18]. Moreover, the incorporation of Nd into a Ni‐based framework causes significant electronic redistribution induced by the obvious electronegativity difference between Nd and Ni, leading to efficient charge transfer for optimizing intermediate adsorption behaviors. Depending on this proposed synergistic mechanism in which Nd and Ni act as cooperative roles, it was revealed that the optimal catalyst delivered a potential as low as 1.347 V to achieve 10 mA cm^−2^ and an operation voltage of only 1.398 V in the asymmetrical cell. Moreover, when the optimal catalyst was used for Zn‐urea battery, the peak power density reaches 11.6 mW cm^−2^. It is expected that this work provides a mechanistic understanding of Lewis‐acid engineering for manipulating multi‐electron reaction pathways and offers a promising pathway for renewable energy conversion and sustainable environmental remediation.

## 2 Results and Discussion

**FIGURE 1:**
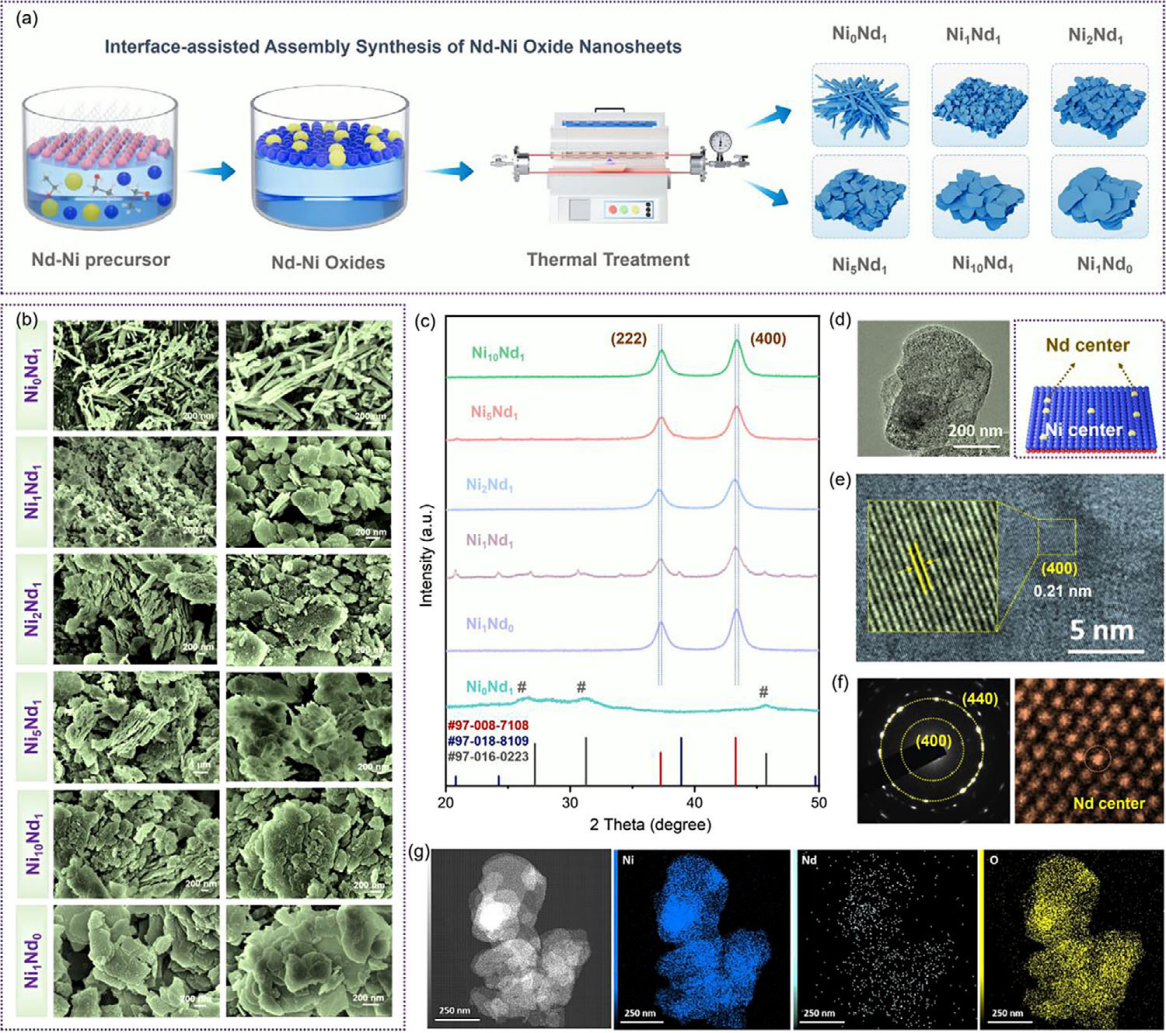
Morphological and structural characterizations of Nd‐Ni oxide catalysts. (a) Schematic illustration of the fabrication of Nd‐Ni oxide catalysts. (b) SEM images and (c) XRD patterns of a series of Nd‐Ni oxide catalysts. (d) TEM image and schematical illustration of Nd‐Ni centers on catalyst surfaces, (e) high‐resolution transmission electron microscope (HRTEM) image, and (f) selected area electron diffraction (SAED) pattern of the Ni_5_Nd_1_ catalyst. (g) High‐angle annular dark‐field TEM (HAADF‐TEM) image and element mapping patterns of the Ni_5_Nd_1_ catalyst.

Based on the design concept on Nd‐Ni cooperative centers, a series of Nd‐Ni oxide catalysts with different Nd/Ni ratios were rationally synthesized by an interface‐assisted hydrothermal reaction coupled with thermal treatment, as illustrated in Figure 1a. Specifically, Ni and Nd‐containing salt precursors were dissolved into a water‐ethanol system in the presence of sodium acetate (CH_3_COONa), and then thermally treated at 180°C for 12 h. After annealed at 350°C with a ramping rate of 5°C min^−1^ in a flowing nitrogen atmosphere for 2 h, a series of Nd‐Ni oxide catalysts with different Nd/Ni ratios were produced. Based on the different Ni‐Nd precursor ratios of 0:1, 1:1, 2:1, 5:1, 10:1, and 1:0, the corresponding samples were labeled as Ni_0_Nd_1_, Ni_1_Nd_1_, Ni_2_Nd_1_, Ni_5_Nd_1_, Ni_10_Nd_1_, and Ni_1_Nd_0_, respectively. It is expected to demonstrate that this unique Nd‐Ni interaction is a powerful design for restructuring UOR process and improving catalytic activity.

First of all, morphological and structural characterizations of a series of Nd‐Ni oxide catalysts were conducted by means of scanning electron microscope (SEM) and powder X‐ray diffraction (XRD). As compared in Figure 1b, only in the presence of Nd center, the Ni_0_Nd_1_ catalyst exhibits a bundle‐like morphology consisting of rods with the length up to micrometer (Figure S1). In the contrast, if without Nd center, the Ni_1_Nd_0_ catalyst demonstrates a 2D nanosheet‐like morphology (Figure S2). When the co‐existence of Nd and Ni centers, as the percentage of Ni center decreases, from the Ni‐rich Ni_10_Nd_1_ catalyst to the Ni‐deficient Ni_1_Nd_1_ catalyst, the 2D morphologies are similar, however, the planer size becomes smaller and smaller (Figures S3–S6). Then, Figure 1c shows the XRD patterns of a series of Nd‐Ni oxide catalysts, which indicates that the cubic NiO (Fm‐3m, PDF#97‐008‐7108) and the monoclinic Nd_2_O_3_ (26.9°, 30.6°, and 45.6°; C2/m, PDF#97‐016‐0223) phases are identified in the Ni_1_Nd_0_ and Ni_0_Nd_1_ catalysts, respectively. For other Nd‐Ni oxide catalysts with different Nd/Ni ratios, including Ni_1_Nd_1_, Ni_2_Nd_1_, Ni_5_Nd_1_, Ni_10_Nd_1_, two dominant peaks located at ≈37° and ≈43° belong to (222) and (400) planes for the NiO phase and also a trace of hexagonal NdNi_3_H_3.9_ phase (20.8°, 24.2°, and 38.8°; R‐3m, PDF#97‐018‐8109) is observed, suggesting that Nd centers are primarily incorporated into NiO framework. Moreover, the typical peaks in XRD shift to lower angels for enlarged crystal planes from the Nd‐deficient Ni_10_Nd_1_ catalyst (37.3°) to the Nd‐rich Ni_1_Nd_1_ catalyst (37.2°), indicating the successful implantation of high‐radius Nd centers into NiO structure. To accurately confirm Nd‐doping concentration, inductively coupled plasma‐optical emission spectrometer (ICP‐OES) measurements were carried out for a series of Nd‐Ni oxide catalysts (Table S1), which concluded that the Nd‐rich Ni_1_Nd_1_ catalyst showed the highest Nd ratio of 38.81 wt.% while the Nd‐deficient Ni_10_Nd_1_ catalyst presented only the lowest value of only 0.06 wt.%, and the Ni_2_Nd_1_ and Ni_5_Nd_1_ catalysts showed medium Nd ratios of 2.19 wt.% and 0.60 wt.%, respectively.

Then, transmission electron microscope (TEM) was utilized to reveal more structural details on the resultant catalyst. As shown in Figure 1d, a typical sheet‐like morphology is identified for the Ni_5_Nd_1_ catalyst (Figure S7), and the primary phase is the NiO framework, as verified by the lattice spacing of 0.21 nm corresponding to the (400) plane in high‐resolution TEM (HRTEM) image (Figure 1e) and the (400)/(440) patterns in the selected area electron diffraction (SAED) pattern (Figure 1f), in the presence of a trace of NdNi_3_H_3.9_ phase (Figure S8). Besides, high‐angle annular dark‐field TEM (HAADF‐TEM) image and the corresponding element mapping patterns (Figure 1g) evidences the uniform element distribution of Nd, Ni and O in the Ni_5_Nd_1_ catalyst (Figure S9), implying the homogeneous Nd doping in the NiO structure.

**FIGURE 2:**
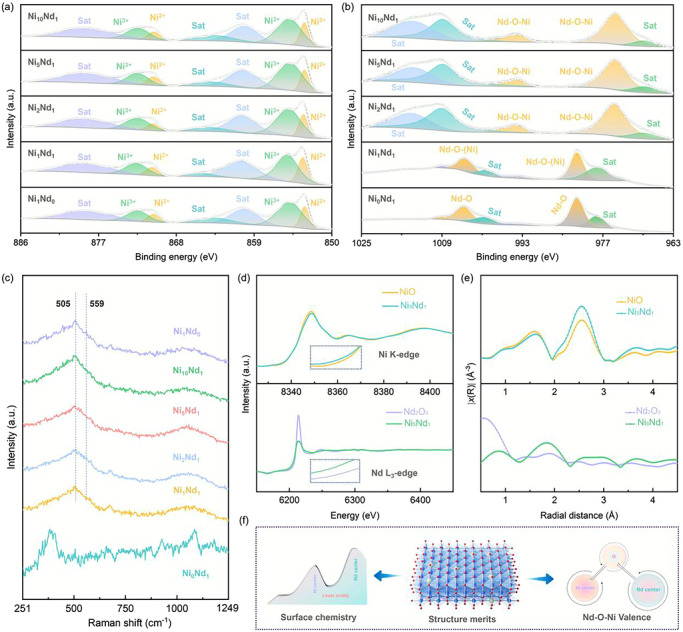
Morphological and structural characterizations of Nd‐Ni oxide catalysts. (a,b) High‐resolution XPS spectra of (a) Ni 2p and (b) Nd 3d in a series of Nd‐Ni oxide catalysts. (c) Raman spectra of a series of Nd‐Ni oxide catalysts. (d) X‐ray absorption near‐edge structure (XANES) spectra at Ni K‐edge and Nd L‐edge, and (e) the corresponding Fourier‐transform edge extended X‐ray absorption fine structure (EXAFS) spectra of the Ni_5_Nd_1_ catalyst and the reference samples. (f) Schematical illustration of structure merits of Nd‐Ni oxide catalysts.

X‐ray photoelectron spectroscopy (XPS) technique was further employed to examine surface chemistry of these resultant Nd‐Ni oxide catalysts. Both Nd and Ni are clearly identified in the survey spectra of the Nd‐Ni oxide catalysts (Figures S10 and S11). As compared in the fitted Ni 2p XPS spectra (Figure 2a), in the Ni_1_Nd_0_ catalyst, two pairs of representative peaks located at 853.15/870.62 and 854.87/872.45 eV are ascribed to Ni^2+^ (2p_3/2_/2p_1/2_) and Ni^3+^ (2p_3/2_/2p_1/2_), respectively [10, 19]. As the percentage of Nd increases from the Ni_0_Nd_1_ to Ni_1_Nd_1_, the dominate peak for Ni 2p_3/2_ gradually shifts from the initial 853.18 eV to a high energy of 853.36 eV, indicating the changes on hybrid orbits of Ni‐O bonds after the incorporation of Nd centers. For Nd 3d XPS spectra (Figure 2b), a couple of obvious signals appeared at 982.20 and 1004.69 eV are attributed to 3d_5/2_ and 3d_3/2_, respectively, for Nd^3+^ in the Ni_0_Nd_1_ catalyst. In the contrast to Ni, as the percentage of Nd decreases from the Ni_0_Nd_1_ to Ni_1_Nd_1_, the binding energy for Nd 3d_5/2_ gradually decreases from the initial 974.57 to 982.17 eV, which implies the electronic interaction of Nd‐O‐Ni bonding in Nd‐Ni oxide catalysts [20]. Subsequently, Raman spectra are compared in Figure 2c. For the Ni_0_Nd_1_ catalyst without Ni centers, these typical Raman peaks are found to be attributed to Nd_2_O_3_ [21]. For the Ni_1_Nd_0_ catalyst without Nd centers, the peak located at around 505 cm^−1^ was attributed to phonon‐magnon excited scattering at the Brillouin zone center [22], and the other scattering signal at approximately 559 cm^−1^ is related to the longitudinal mode of Ni‐O bonds, suggesting the formation of both Ni^2+^‐O and Ni^3+^‐O species [23]. In the coexistence of Nd and Ni centers, as the Ni ratio decreases, the Raman signals for Ni^3+^‐O became relatively weaker, which indicates that the incorporation of Nd centers could accommodate the surface oxidation of Ni centers into high‐valence ones. Finally, X‐ray absorption near‐edge structure (XANES) spectra at Ni K‐edge and Nd L_3_‐edge, as demonstrated in Figure 2d, the Ni_5_Nd_1_ catalyst delivers a low pre‐edge energy in comparison to NiO and Nd_2_O_3_, indicating that the Ni‐O‐Nd bonding is lower than that of the Ni‐O and Nd‐O bonding, thus inducing structural stability for Nd‐Ni interaction. Also, based on the Fourier‐transform edge extended X‐ray absorption fine structure (EXAFS) spectra (Figure 2e), the local environment of both Ni‐O and Ni‐Ni bonds are changed in the presence of Nd centers, and the newly formed Nd‐O‐Ni bond is completely different to Nd‐O in the Nd_2_O_3_.

Based on above‐mentioned analysis, as depicted in Figure 2f, the Nd‐Ni oxide catalysts manifest the unique Nd‐O‐Ni interaction, which is crucial for restructuring UOR and improving catalytic activity. Also, owing to the stronger Lewis acidity of the introduced Nd centers than that of Ni centers, the resultant Nd‐Ni oxide catalysts exhibit more acidic sites, which are expected to be favorable for urea adsorption behaviors.

**FIGURE 3:**
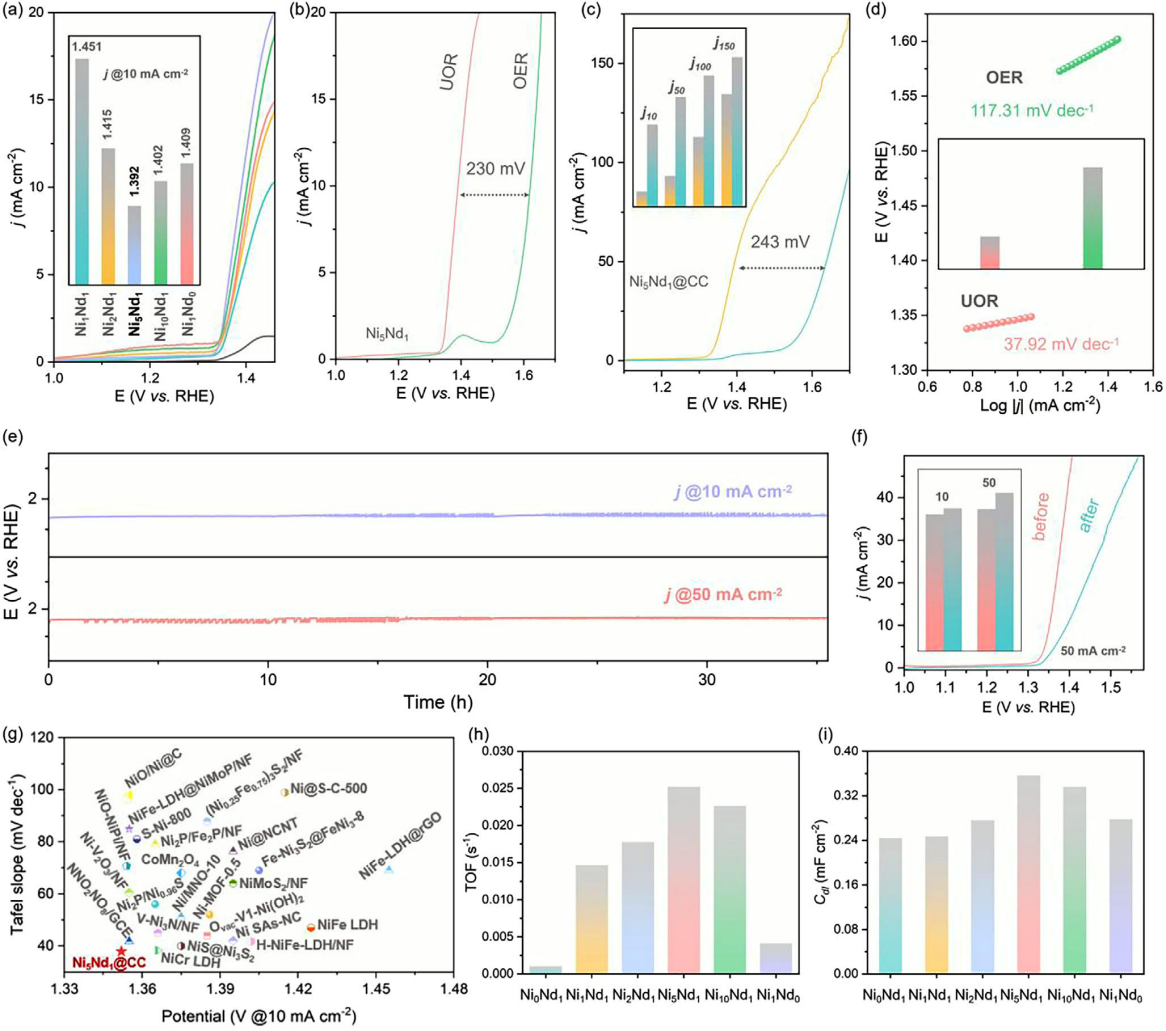
Electrocatalytic properties of a series of Nd‐Ni oxide catalysts for UOR and OER under alkaline solution. (a) iR‐corrected linear sweep voltammetry (LSV) plots and potential comparison of a series of Nd‐Ni oxide catalysts at 10 mA cm^−2^ for UOR in 0.33 M urea + 1.0 M KOH solution. (b) iR‐corrected LSV plots of the Ni_5_Nd_1_ catalyst for UOR and OER. (c) iR‐corrected LSV plots and potential comparison of the Ni_5_Nd_1_@CC catalyst at 10 mA cm^−2^ for UOR and OER. (d) Tafel slope of the Ni_5_Nd_1_@CC catalyst for UOR and OER. (e) Chronopotentiometric measurement of the Ni_5_Nd_1_@CC catalyst at 10 and 50 mA cm^−2^. (f) LSV plots and potential comparison of the Ni_5_Nd_1_@CC catalyst before and after stability tests. (g) Comparison on catalytic activities of the Ni_5_Nd_1_@CC catalyst with these previously reported catalysts under alkaline condition. (h) Comparison on TOF of Nd‐Ni oxide catalysts. (i) Comparison on C*_dl_* values of Nd‐Ni oxide catalysts.

Electrocatalytic UOR performance of the resulting Ni‐Nd oxide catalysts were evaluated using a three‐electrode system in a urea‐containing alkaline solution and OER properties using the urea‐free solution were also assessed. As compared on the linear sweep voltammetry (LSV) plots of Nd‐Ni oxide catalysts for UOR (Figure 3a) and OER (Figure S12), the Ni_5_Nd_1_ catalyst loaded onto glassy carbon electrode (GCE) delivered the lowest potential of 1.392 V for UOR and 1.622 V for OER, respectively, to generate the required density of 10 mA cm^−2^. Without Ni center, the Ni_0_Nd_1_ catalyst exhibited nearly no activity toward OER and UOR. Without Nd center, the Ni_1_Nd_0_ catalyst presented potentials of 1.409 V for UOR and 1.661 V for OER. If the co‐existence of Nd and Ni centers, the Ni_1_Nd_1_ and Ni_2_Nd_1_ catalysts containing high‐concentration Nd centers are inferior to the Ni_1_Nd_0_ catalyst without Nd center, however, the Ni_5_Nd_1_ and Ni_10_Nd_1_ catalysts with low‐concentration Nd centers showed better performance, particularly the Ni_5_Nd_1_ catalyst manifested the best performance. These results imply that the cooperative roles of Nd and Ni centers are highly dependent on the relative concentration. High‐concentration Nd centers might seriously distort the Ni‐O framework and lead to the Nd aggregation for possible phase formation, resulting in the loss of active sites, while low‐concentration Nd centers could well tune surface chemistry states of Ni‐O structure, promote the uniform distribution of active sites, and increase structural stability. It should be noteworthy that the synergistic effect could not well amplified if the concentration of Nd centers is too low, as identified in the Ni_10_Nd_1_ catalysts with only 0.06 wt% Nd. Furthermore, the UOR is highly active in comparison to OER, in which a potential gap of 230 mV was identified for the Ni_5_Nd_1_ catalyst (Figure 3b). If carbon cloth (CC) was used as the substrate (Figure 3c), the potential was greatly reduced to 1.347 and 1.395 V at 10 and 50 mA cm^−2^, respectively, using the Ni_5_Nd_1_@CC catalyst, which was much smaller than that for OER (1.553 V at 10 mA cm^−2^ and 1.638 V at 50 mA cm^−2^). Even at a high density of 150 mA cm^−2^, the potential for the Ni_5_Nd_1_@CC catalyst was 1.647 V.

To evaluate the reaction kinetics, Tafel slopes for UOR and OER were calculated for a series of Nd‐Ni oxide catalysts, as demonstrated in Figures S13 and S14. Based on the GCE electrode, the Tafel slope followed an order as Ni_1_Nd_1_> Ni_2_Nd_1_> Ni_1_Nd_0_> Ni_10_Nd_1_> Ni_5_Nd_1_ for UOR (Figure S15), however, the order for OER was different (Ni_10_Nd_1_> Ni_1_Nd_0_> Ni_1_Nd_1_> Ni_2_Nd_1_> Ni_5_Nd_1_) (Figure S16), which means that Nd‐Ni interaction is crucial for reaction kinetics, especially for UOR, and the Ni_5_Nd_1_ catalyst demonstrated the best kinetics for both UOR and OER. After loaded onto the CC substrate, the resultant Ni_5_Nd_1_@CC catalyst showed a quite low slope of 37.92 mV dec^−1^, suggesting a most favorable UOR kinetic compared to OER with a high value of 117.31 mV dec^−1^ (Figure 3d). Chronopotentiometric measurements on the Ni_5_Nd_1_@CC catalyst were conducted for continuous over 35 h at densities of 10 and 50 mA cm^−2^ (Figure 3e), and the UOR potential increased by 4.43% and 11.37% at 10 and 50 mA cm^−2^, respectively (Figure 3f), suggesting good structural stability.

According to the electrochemical results, the optimal Ni_5_Nd_1_@CC electrocatalyst manifests better activity toward UOR than OER. Moreover, as compared in Figure 3g, the Ni_5_Nd_1_@CC catalyst exhibits low potential and Tafel slope than other Ni/Co‐based catalysts (Table S2), such as NiFe LDH [24], NiO/Ni@C [25], and O*_v_*/V‐doped Ni(OH)_2_ [26]_._ This is largely associated with the additional active sites contributed by Nd centers, which cooperate with Ni centers to promote UOR activity. Turnover frequency (TOF) was calculated based on Nd and Ni centers (Figure S17) and the Ni_5_Nd_1_ catalyst exhibited the highest value of 2.52 × 10^−2^ s^−1^ at 1.45 V, which is much larger than that for the Ni_0_Nd_1_ (1.03 × 10^−3^ s^−1^) and the Ni_1_Nd_0_ (4.12 × 10^−3^ s^−1^) catalysts (Figure 3h). Besides, electrochemical double‐layer capacitance (C*_dl_*) as an indicator of electrochemical active surface area (ECSA) was estimated using cyclic voltammetry (CV) technique (Figure S18). By plotting the linear relations between scan rate and current density, the *C*_dl_ values were obtained through slope calculation (Figure S19), which concluded that the Ni_5_Nd_1_ catalyst exhibited the highest *C*_dl_ value of 0.357 mF cm^−2^ (Figure 3i).

**FIGURE 4:**
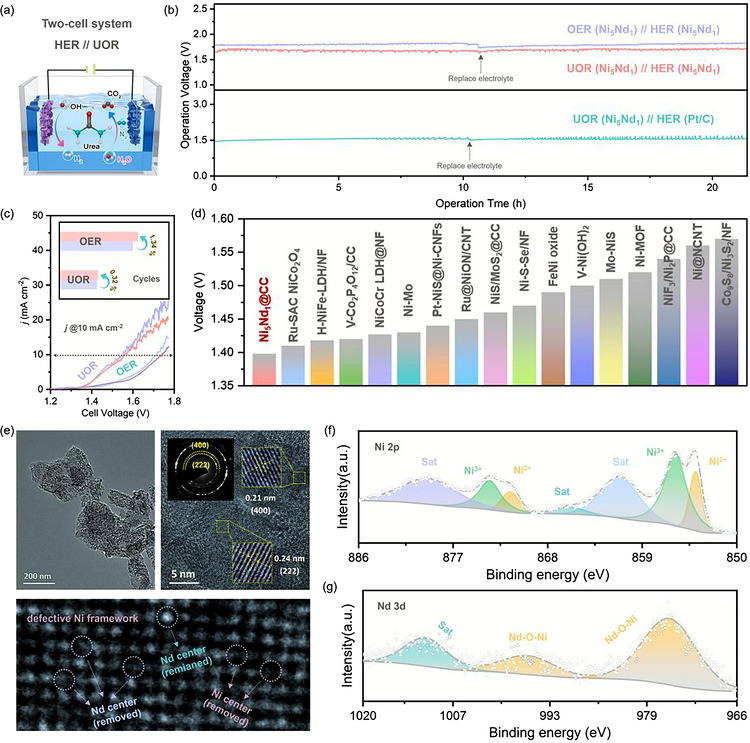
Electrochemical performance of two‐cell system assembled by the resultant Ni_5_Nd_1_@CC catalyst for UOR and HER in the alkaline solution. (a) Schematic illustration of reaction pathways in the two‐electrode cell for UOR and HER. (b) Chronopotentiometric measurement of the Ni_5_Nd_1_@CC catalyst based two‐electrode symmetrical and asymmetrical (coupled with Pt/C) systems for UOR/OER and HER. (c) LSV plots before and after chronopotentiometric measurements and the corresponding operation voltages. (d) Comparison of operation potentials of the Ni_5_Nd_1_@CC‐based cell with these previously reported systems. (e) TEM and HRTEM images, and (f,g) high‐resolution XPS spectra of Ni 2p and Nd 3d for the Ni_5_Nd_1_@CC catalyst after long‐term stability measurements under alkaline condition.

To verify the practical potential, as shown in Figure 4a, a two‐cell system was assembled using the resultant Ni_5_Nd_1_@CC catalyst for UOR//HER in the alkaline solution. For comparison, the symmetrical cells for OER//HER and the asymmetrical cell by coupled with Pt/C were assembled for chronopotentiometric measurements. As presented in Figure 4b, the voltage of the Ni_5_Nd_1_@CC catalyst based symmetrical cell for UOR//HER is constantly lower than that for OER//HER during continuous operation for over 20 h under alkaline conditions. Furthermore, as shown in Figure 4c, the operating voltage for the UOR//HER system increases by only 0.32% after long‐term cycles, which is much smaller than that for OER//HER (1.34%). If the commercialized Pt/C catalyst is paired with the Ni_5_Nd_1_@CC catalyst to form an asymmetrical cell, a much lower operation voltage of 1.398 V can be achieved., suggesting great potential for large‐scale application. Compared with these reported Ni‐containing catalysts based two‐cell systems (Table S3), the operation voltage is much lower than that of Ni‐containing catalysts based two‐electrode systems, such as Ni‐Mo [27], Mo‐NiS [28], and P‐CoNi_2_S_4_ [29] (Figure 4d). In addition, the variety on the *R*_ct_ value in EIS spectra (Figure S20) after UOR//HER (55.58 ohm) are inapparent compared to that for OER//HER (65.31 ohm) (Figure S21) in the symmetrical cells, and a much lower value (35.85 ohm) was identified in the asymmetrical cell (Figure S22), implying the favorable stability for UOR of the Nd‐Ni oxide catalyst.

Structural characterizations were conducted on the Ni_5_Nd_1_ catalyst to evidence the possible change after long‐time stability tests. Figure 4e shows the TEM and HRTEM images, concluding that the 2D sheet‐like morphology and the dominate NiO framework remains (Figure S23), however, a defective surface is obtained due to the partial removal of Nd and Ni centers over cycles (Figure S24). Also, high‐resolution XPS spectra of Ni 2p (Figure 4f) and Nd 3d (Figure 4g) shows the oxidization of Ni centers as identified by the highest energy of 853.92 eV and the loss of Nd centers as observed by the low Nd‐signal intensity and the widened Nd‐O‐Ni peak in XPS. Besides, when the Ni_5_Nd_1_ catalyst was immersed in different KOH solutions with various concentrations (Figure S25), as the concentration increases, the UOR activity progressively decreases, suggesting the existence of vacancies for supporting the activity.

**FIGURE 5:**
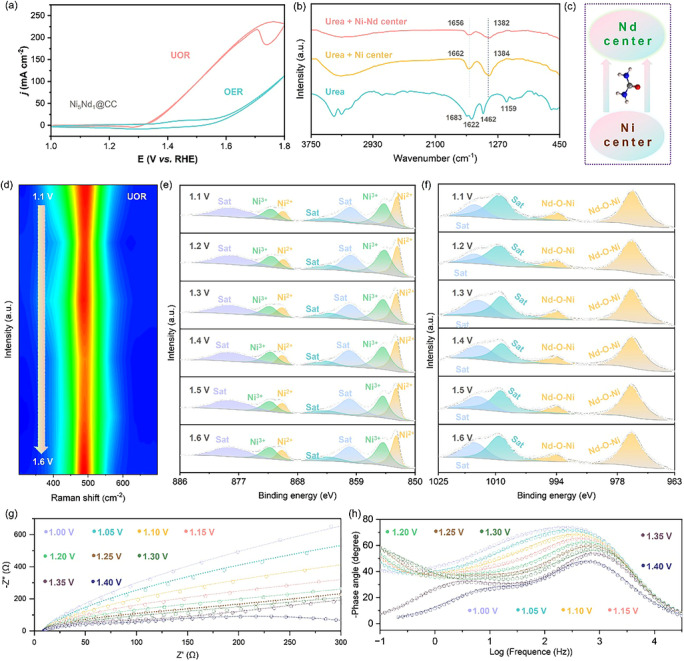
Electrocatalytic enhanced mechanism of the Ni_5_Nd_1_ catalyst toward UOR under alkaline condition. (a) CV curves of the Ni_5_Nd_1_@CC catalyst for UOR and OER. (b) FT‐IR spectra of urea molecules and urea‐adsorbed Ni or Nd‐Ni centers. (c) Schematical illustration of Nd‐Ni cooperative roles for UOR. (d) Potential‐dependent Raman spectra of the Ni_5_Nd_1_ catalyst for alkaline UOR. (e,f) Potential‐dependent XPS spectra at (e) Ni 2p and (f) Nd 3d of the Ni_5_Nd_1_ catalyst for alkaline UOR. (g) EIS spectra and (h) Bode phase plots of the Ni_5_Nd_1_ catalyst for UOR at a potential range between 1.00 and 1.40 V.

To elucidate the possible catalytic enhanced mechanism of the Ni_5_Nd_1_ catalyst toward UOR, as verified by the significantly larger densities for UOR than that for OER (Figure 5a), the structural varieties during UOR were tracked by spectroscopy techniques. First, Fourier transform infrared spectroscopy (FT‐IR) spectra on the free urea molecules and the urea adsorbed onto Ni (Ni_1_Nd_0_) or Nd‐Ni (Ni_5_Nd_1_) centers were compared (Figure 5b). The wavenumber shifts for these peaks belonging to urea in urea‐adsorbed Ni or Nd‐Ni centers confirmed the chemical bonding between urea and catalysts. Moreover, compared to urea adsorbed onto Ni center (1662 and 1384 cm^−1^), these peaks appeared at lower wavenumber (1656 and 1382 cm^−1^) for urea adsorbed onto Nd‐Ni centers. Combined with the changes on N 1s, Ni 2p and Nd 3d in XPS (Figure S26) and the symmetric N–C–N stretching mode in Raman (Figure S27), it is suggested that the increased interaction between urea and metal centers in the presence of Nd centers and the competitive adsorption behaviors between Nd and Ni centers (Figure 5c). Then, Raman and XPS techniques were applied to track the underlying structural varieties on surface chemistry of the Ni_5_Nd_1_ catalyst during UOR at a potential range from 1.1 to 1.6 V. As shown in Figure 5d, a small shoulder peak around 465 cm^−1^ observed from 1.1 to 1.6 V (Figure S28) was assigned to the formation of the low‐crystalline NiOOH species [30, 31]. Compared to the change for the Ni_1_Nd_0_ catalyst without Nd center, in which a sharp peak appeared as the indicator of NiOOH species (Figure S29), the formation of NiOOH was partially inhibited in the Ni_5_Nd_1_ catalyst, indicating the crucial role of Nd center for supporting UOR. To summarize, the sharp Raman signals originate from the vibration of chemical bonds in a uniform environment with the nearly identical Ni sites, which indicates the formation of a well‐crystallized and structurally uniform NiOOH phase in the Ni_1_Nd_0_ catalyst. On the contrast, in the presence of Nd center, the broad shoulder peaks were identified, which suggests the formation of a poorly crystalline and structurally disordered NiOOH phase in the Ni_5_Nd_1_ catalyst. Owing to the much larger ionic radius of Nd‐ions than that of Ni‐ions, the incorporation of Nd centers could induce structural disorder and lattice strain. During UOR, this existing disorder prevents the formation of a long‐range and ordered NiOOH structure, however, it favors the appearance of a disordered and defect‐rich nano‐crystalline NiOOH phase. Then, Nd centers can modify the electron density around neighboring Ni atoms, which potentially facilitates the formation of Ni(III) [32], thus leading to a mixed‐valence oxyhydroxide surface. Moreover, the co‐existence of Nd and Ni centers could produce heterogeneous active sites, which could transform into active phases for producing the Nd‐Ni synergistic effect.

Based on Ni 2p XPS spectra (Figure 5e) that were captured at a potential range between 1.00 and 1.40 V during UOR, the calculated Ni(III)/Ni(II) ratios for the Ni_5_Nd_1_ catalyst nearly remain unchanged from 1.1 to 1.6 V, and only slight positive shifts were identified for Ni(III)‐O and Ni(II)‐O signals, which suggests the stable Ni sites on the surface. By comparison, the shift on Nd‐O‐Ni was obvious from 973.88 eV in 1.1 V to 974.16 eV in 1.6 V (Figure 5f). These results indicate that Nd centers as the synergistic sites with Ni centers are favorable to support UOR, accompanied by the surface oxidation behaviors during UOR. In addition, EIS was applied to examine the interface charge transfer of the Ni_5_Nd_1_ catalyst during UOR. As the potential increases from 1.00 to 1.40 V, the diameter of the semicircle region (Figure 5g) and the phase angle peak of the low‐frequency region in the Bode plots (Figure 5h) at different UOR potentials decreases, which suggests a favorable charge transfer to modulate the intermediate adsorption behaviors for promoting UOR [33, 34, 35, 36].

**FIGURE 6:**
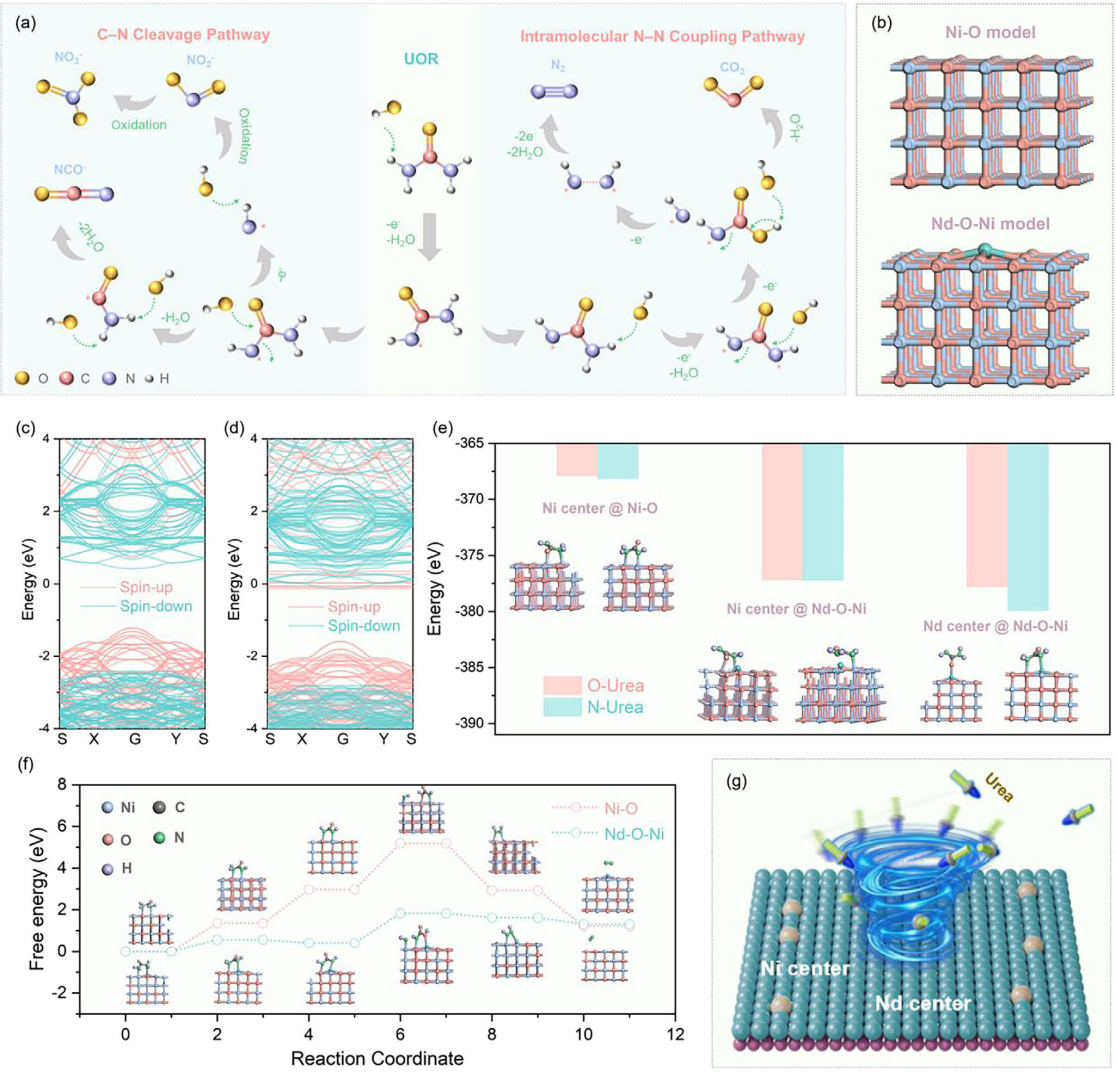
Theoretical analysis on electrocatalytic mechanism of Nd‐Ni oxide catalyst for UOR. (a) Schematic illustration of reaction pathways for UOR. (b) Theoretical models of the Ni‐O and Nd‐O‐Ni models. (c,d) Band structures of Ni‐O and Nd‐O‐Ni models. (e) Comparison on energies of urea molecules and hydroxyl group adsorbed onto Ni and Nd centers in Ni‐O and Nd‐O‐Ni models. (f) Intermediate adsorption energies along different UOR pathways of the Nd‐O‐Ni model. (g) Schematic illustration of structural merits of the Nd‐Ni oxide catalyst for UOR.

Theoretical calculation was conducted to further validate the design concept on a synergistic environment in the presence of both Nd and Ni centers for enhancing the adsorption and activation of the urea molecule. First, to confirm the possible pathway, the electrolyte after long‐term stability test was analyzed using ion chromatography (Table S4), in which CO_3_^2−^, HCO_3_^2−^, NO_2_^−^, NO_3_^−^, and OCN^−^ groups were identified, which suggests the co‐existence of C‐N cleavage and intramolecular N‐N coupling pathways, as illustrated in Figure 6a. Then, the Ni‐O and Nd‐O‐Ni models were built (Figure 6b) and electronic structures were examined via calculating the density of states (DOS) and analyzing the band structure. By comparison, the Ni‐O model exhibits a semiconductor‐like surface (Figure S30) with a bandgap of 1.37 eV (Figure 6c), however, the Nd‐O‐Ni model exhibits a metallic character after the incorporation of Nd centers (Figure 6d and Figure S31), indicating the strong Nd‐O‐Ni interaction. It can be inferred that the presence of Nd centers causes significant electronic redistribution and local environment that was induced by the obvious electronegativity difference between Nd and Ni [37], leading to efficient charge transfer for optimizing intermediate adsorption [38]. Subsequently, to evidence the adsorption capability of Nd and Ni sites, the oxygen and nitrogen edges of the urea molecular were adsorbed onto Ni centers and Nd centers in the Ni‐O and Nd‐O‐Ni models, as illustrated in Figure 6e, the most stable model structure is the one consisting of urea‐adsorbed onto Nd sites, which clearly confirm the Nd and Ni centers as dual active sites for efficiently catalyzing UOR. Furthermore, the reaction intermediate adsorption energies along UOR pathways were calculated for the Ni‐O and Nd‐O‐Ni models (Figure 6f). It can be concluded that the strong Lewis acid Nd centers preferentially adsorb and activate the amine (–NH_2_) group of urea, and the dual‐site activation greatly lowers the energy barrier for the critical C–N cleavage and N‐N coupling pathways in UOR [39].

Hence, the structural characteristics are typically associated with attractive UOR catalytic activity for several reasons, which can be summarized as follows (Figure 6g): i) the unique Nd‐O‐Ni interaction is crucial for improving catalytic activity; ii) the introduced Nd centers manifest the stronger Lewis acidity, which offers more acidic sites; iii) the disordered and defect‐rich structure provides a higher number of unsaturated sites and oxygen vacancies, which are highly active for catalytic reactions; iv) the heterogeneous electronic environment indicates a wider range of adsorption strengths, and this diversity could ensure the near‐optimal adsorption energy for urea molecules and various reaction intermediates; v) the defects and mixed valence states can improve electrical conductivity for promoting faster electron transfer during catalytic cycles; vi) the disordered structure can better accommodate the volume changes and stress associated with repeated redox cycles for enhancing long‐term stability.

**FIGURE 7:**
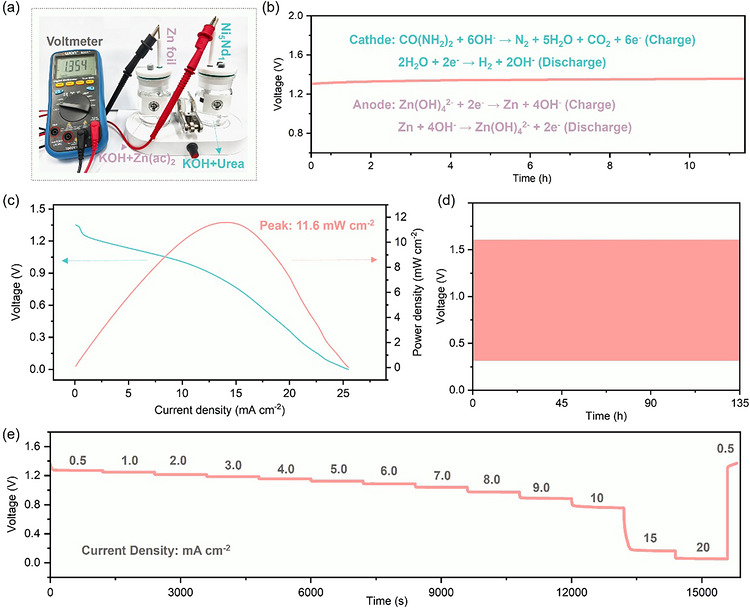
Electrochemical performance of Zn‐urea battery using the Ni_5_Nd_1_@CC catalyst. (a) Photograph of the assembled Zn‐urea battery and the open circuit voltage (OCV) measured using voltmeter. (b) Plots of OCV vs. operation time and reaction equations on cathode and anode. (c) Discharge polarization curve and the corresponding power density plot. (d) Galvanostatic discharge–charge cycling curves for 135 h at a rate of 5 mA cm^−2^. (e) Plots of discharge voltage vs. operation time at different current densities.

Finally, a Zn‐urea battery was assembled, in which Ni_5_Nd_1_@CC for UOR and Pt/C for HER were used as cathode, and Zn foil as anode [40], as shown in Figure 7a, and the battery exhibits a high open circuit voltage (OCV) of 1.354 V, which could be well retained for over 10 h (Figure 7b), suggesting the stable operation of the assembled battery. Upon discharge, Zn was oxidized at the anode and water was reduced at the cathode. Upon charge, UOR and the deposition of Zn occur at the cathode and anode, respectively. Depending on the reaction mechanism, the assembled battery manifests a peak power density as high as 11.6 mW cm^−2^ (Figure 7c), and an attractive long‐term operation for continuous 135 h (Figure 7d) at a current density of 5 mA cm^−2^. Moreover, as displayed in Figure 7e, after discharging at different current densities from 0.5 to 20 mA cm^−2^, the voltage can be returned back when the density was changed into the initial 0.5 mA cm^−2^, indicating stable energy output for potential applications.

## 3 Conclusions

In conclusion, the integration of rare‐metal Nd centers to engineer Lewis acid sites together with Ni centers is proposed to overcome the intrinsic limitations of the current Ni‐based UOR catalysts. Electrocatalytic evaluation results indicated that the optimal catalyst exhibited exceptional UOR activity, requiring only 1.347 V to reach 10 mA cm^−2^, which is significantly lower than the potential for OER. This enhanced activity is attributed to the strong Lewis acidity of Nd centers as synergistic active sites that promotes the adsorption and activation of urea molecules, the Nd‐O‐Ni interaction creates a disordered and defect‐rich surface structure for providing a higher density of active sites and improving charge transfer kinetics, and the presence of Nd centers for inducing significant electronic redistribution and optimizing intermediate adsorption. Moreover, the practical utility of this catalyst was validated in a two‐electrode system for energy‐saving hydrogen production and in a Zn‐urea battery, which achieved a power density as high as 11.6 mW cm^−2^ and remarkable operational stability for 135 h. Hence, this work provides a fundamental mechanistic understanding on Lewis‐acid engineering for manipulating multi‐electron reaction pathways in electrocatalysis, and provides an effective dual‐site design principle for developing high‐performance electrocatalysts for simultaneous renewable energy generation and wastewater purification.

## 4 Experimental Section

### 4.1 Synthesis of Nd‐Ni Oxide Catalysts

NiCl_2_ and Nd(NO_3_)_3_·6H_2_O as metal precursors were dissolved in a solvent mixture of 20 mL water and 5 mL ethanol at different Ni‐Nd molar ratios, and the mixture was ultrasonicated for several minutes for promoting dissolution. Subsequently, 0.8 g CH_3_COONa powder were added under vigorous stirring at room temperature for 5 h, resulting in a transparent solution, which was then transferred into a 50 mL Teflon‐lined autoclave and maintained at 180°C for 12 h. When the reaction was finished, the powder was obtained by washed with water or ethanol and dried at 60°C. Next, the obtained powder was annealed at 350°C with a ramping rate of 5°C min^−1^ in a flowing nitrogen atmosphere for 2 h in a tube furnace. Based on the different Ni‐Nd precursor ratios of 0:1, 1:1, 2:1, 5:1, 10:1, and 1:0, the corresponding samples were labeled as Ni_0_Nd_1_, Ni_1_Nd_1_, Ni_2_Nd_1_, Ni_5_Nd_1_, Ni_10_Nd_1_, and Ni_1_Nd_0_, respectively.

## Conflicts of Interest

The authors declare no conflicts of interest.

## Supporting information




**Supporting File**: advs75788‐sup‐0001‐SuppMat.pdf.

## Data Availability

The data that support the findings of this study are available from the corresponding author upon reasonable request.
