# Supplementary material for: Lewis‐Acid Engineering with Neodymium Promoters as Synergistic Nd‐Ni Dual Sites for Enhanced Urea Oxidation
Source: Adv Sci (Weinh). 2026 May 22;13(46):e75788. doi: 10.1002/advs.75788 (PMC13336136; doi:10.1002/advs.75788)
Supplement: Supplementary file 1 [file ADVS-13-e75788-s001.pdf]

Supporting Information

Lewis-Acid Engineering with Neodymium Promoters as Synergistic Nd-Ni Dual Sites for Enhanced Urea Oxidation

Experimental Section

Chemicals and Materials

Nickel chloride ($NiCl_2$, 99%) and sodium acetate (CH_3COONa, 99%) were purchased from Macklin Biochemical Technology. Neodymium nitrate hexahydrate ($Nd(NO_3)_3 \cdot 6H_2O$, 98%) and ethanol (C_2H_5OH, ≥99.7%) were purchased from InnoChem Science Technology. Ultrapure water was purified through a Chonyoo purification system.

Material Characterizations

Crystal structures of Nd-Ni oxide catalysts were investigated by X-ray diffraction (XRD) technique on a German Bruker D2 Phaser diffractometer with a copper radiation at a voltage and a current of 40 kV and 40 mA, respectively. Raman spectra of Nd-Ni oxide catalysts were collected on a Horiba LabRAM HR Evolution spectrometer. Inductively coupled plasma optical emission spectrometry (ICP-OES) measurements were conducted to confirm elemental composition of Nd-Ni oxide catalysts using an Agilent 5800 instrument. X-ray photoelectron spectroscopy (XPS) spectra of Nd-Ni oxide catalysts were analyzed on a Thermo Scientific K-Alpha spectrometer. Morphological and structural information of Nd-Ni oxide catalysts was captured using ZEISS GeminiSEM 360 field-emission scanning electron microscope (FE-SEM) and JEOL JEM-F200 transmission electron microscope (TEM) with an accelerated voltage of 200 kV. Fourier transform infrared spectroscopy (FT-IR) plots were recorded on a Shimadzu IRTracer-100. Ion concentrations in the electrolyte were obtained using a Thermo Scientific Dionex Aquion ion chromatography.

Electrochemical Measurements

Electrocatalytic performance of a series of Nd-Ni oxide catalysts for UOR and OER were evaluated using a standard three-electrode system in 1.0 M KOH + 0.33 M urea and 1.0 M

KOH solution, respectively, and Hg/HgO and graphite rod were selected as the reference and the counter electrode, respectively. All tests were performed on a CHI660e electrochemical workstation, and cyclic voltammetry (CV) tests was condcuted before catalytic measurements. Linear sweep voltammetry (LSV) curves with 95% iR compensation were collected at a scan rate of 5 mV s^{-1}. Electrochemical impedance spectroscopy (EIS) plots were obtained at a frequency range of 0.05-10^5 Hz. Long-term stability was assessed using chronopotentiometry technique. Electrochemical double-layer capacitance (C_{dl}) was determined and calculated based on CV technique in non-Faradaic regions to compare electrochemical active surface area (ECSA). All measurements were performed at room temperature.

Turnover frequency (TOF) was calculated based on the following equation:

$$TOF = \frac{|j|A}{\mathrm{m}Fn}$$

where j represents the current density (mA cm^{-2}); A is the electrode area (cm^2); m stands for the electron number consumed per molecule; F is the Faraday constant (96485 C mol^{-1}); and n is the site number (mmol).

Theoretical Calculation Methods

Theoretical calculations were performed using the Vienna *ab initio* simulation package (VASP) based on density functional theory (DFT) under the projector augmented plane-wave (PAW) method and the generalized gradient approximation (GGA) for exchange-correlation potential. The supercells of 2 × 2 × 2 primitive cells are used, and the vacuum direction was set to be 40 Å to eliminate the adjacent layer interactions. The energy cut-off for plane wave was set to 450 eV, and the Brillouin zone integration was performed using a 4 × 4 × 1 k-mesh. DFT-D3 method of Grimme with zero-damping function was used to describe the adsorption process. All the structures were fully relaxed until the residual energy in iterative solution of the Kohn-Sham equation and the force reached less than 10^{-4}

eV and 10^{-2} eV $Å^{-1}$, respectively.

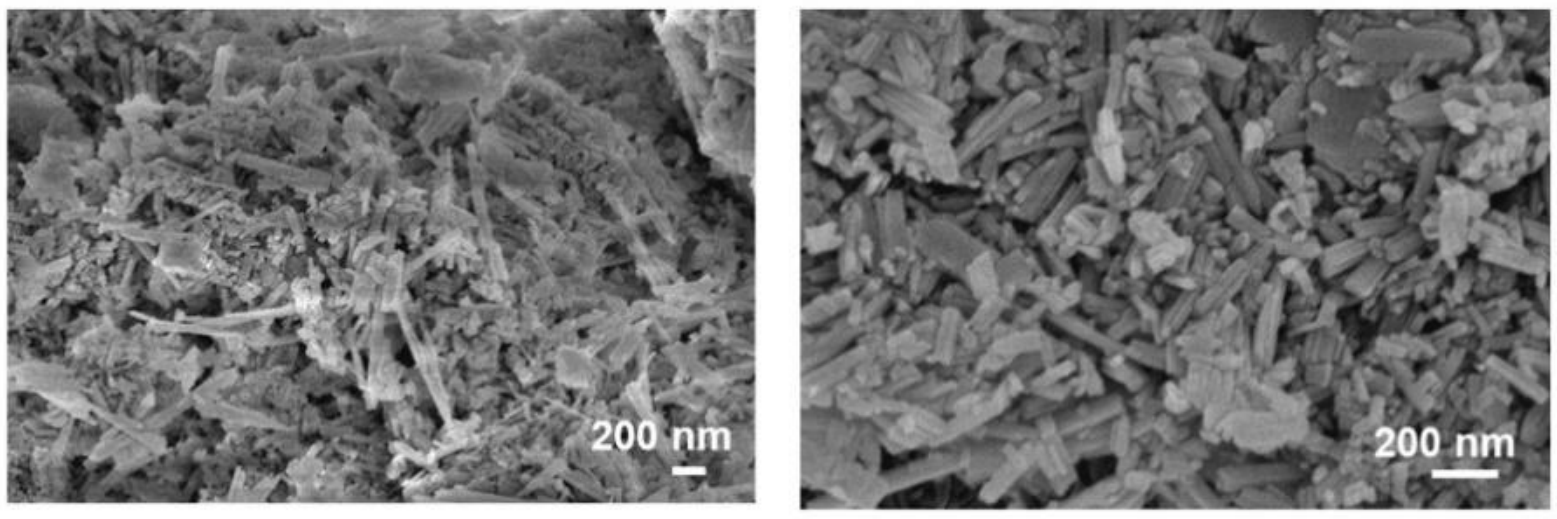

Figure S1. SEM image of the Ni_0Nd_1 catalyst.

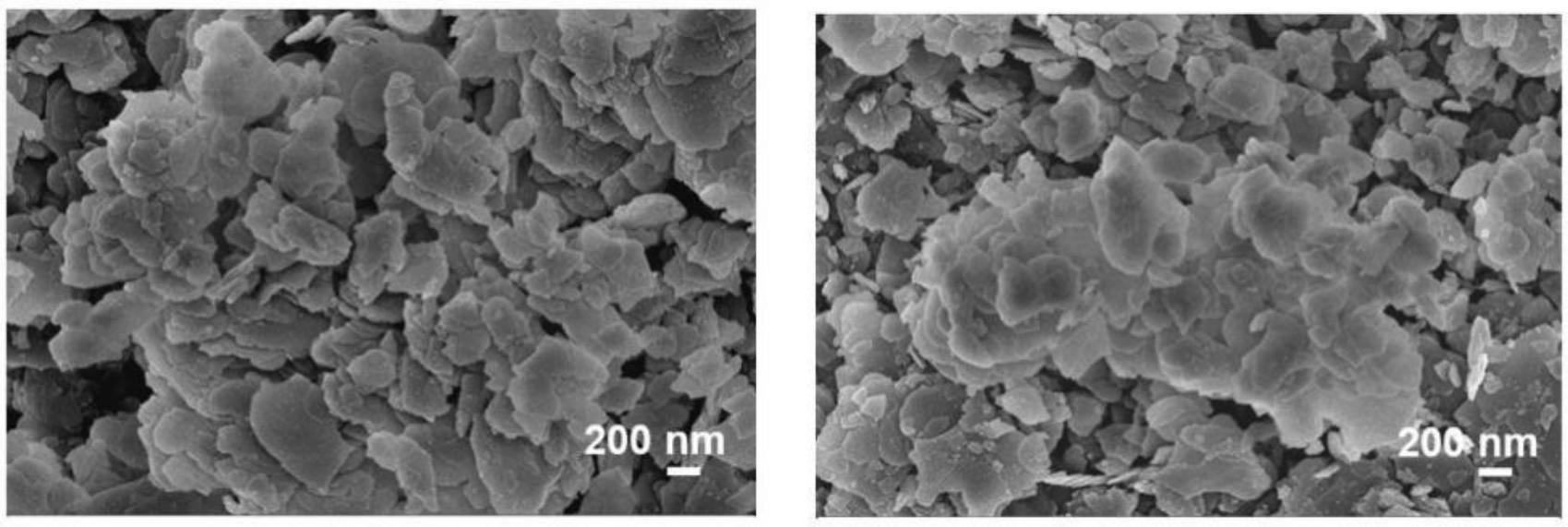

Figure S2. SEM image of the Ni_1Nd_0 catalyst.

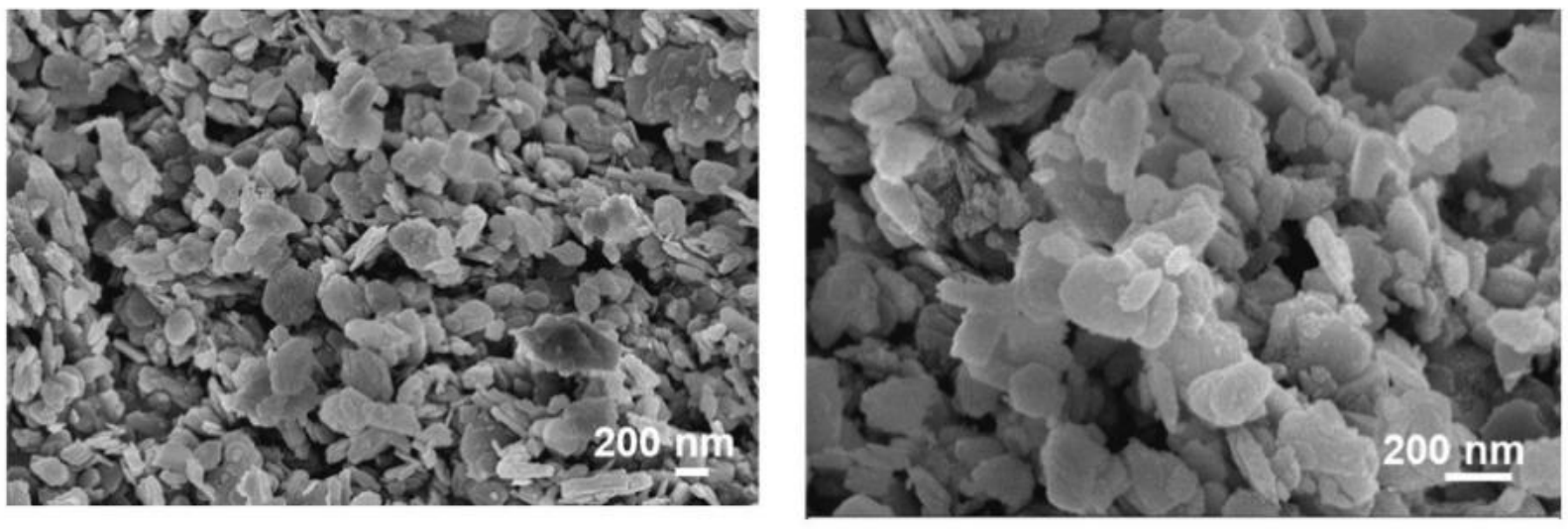

Figure S3. SEM image of the Ni_1Nd_1 catalyst.

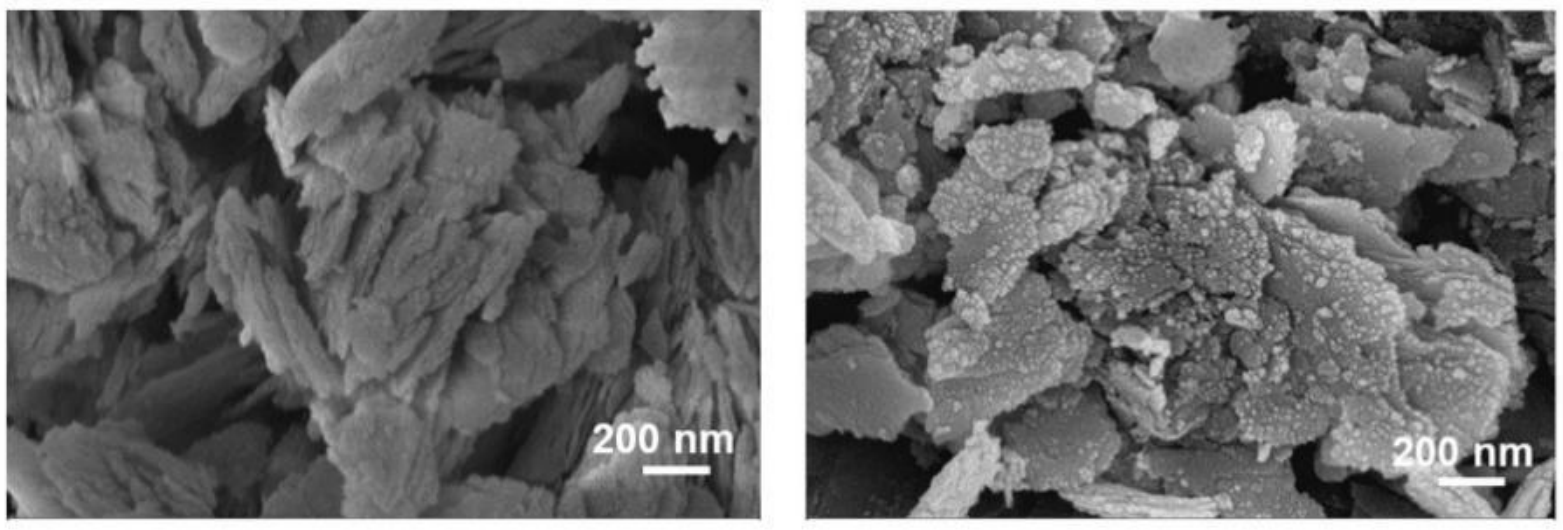

Figure S4. SEM image of the Ni_2Nd_1 catalyst.

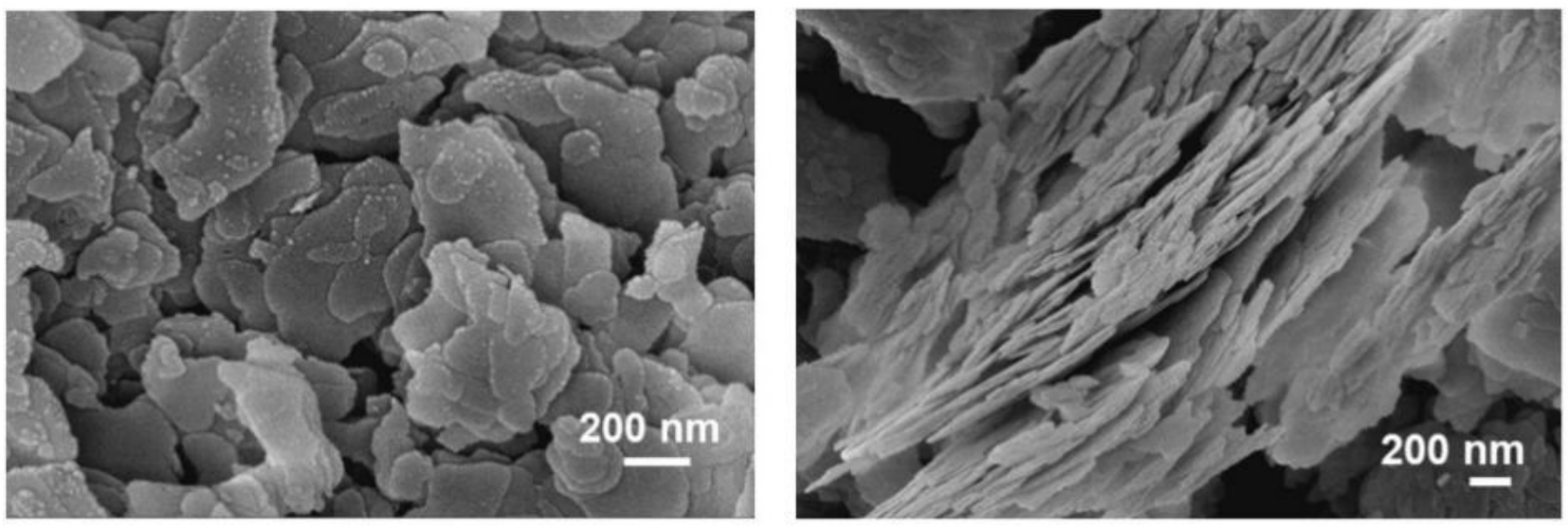

Figure S5. SEM image of the Ni_5Nd_1 catalyst.

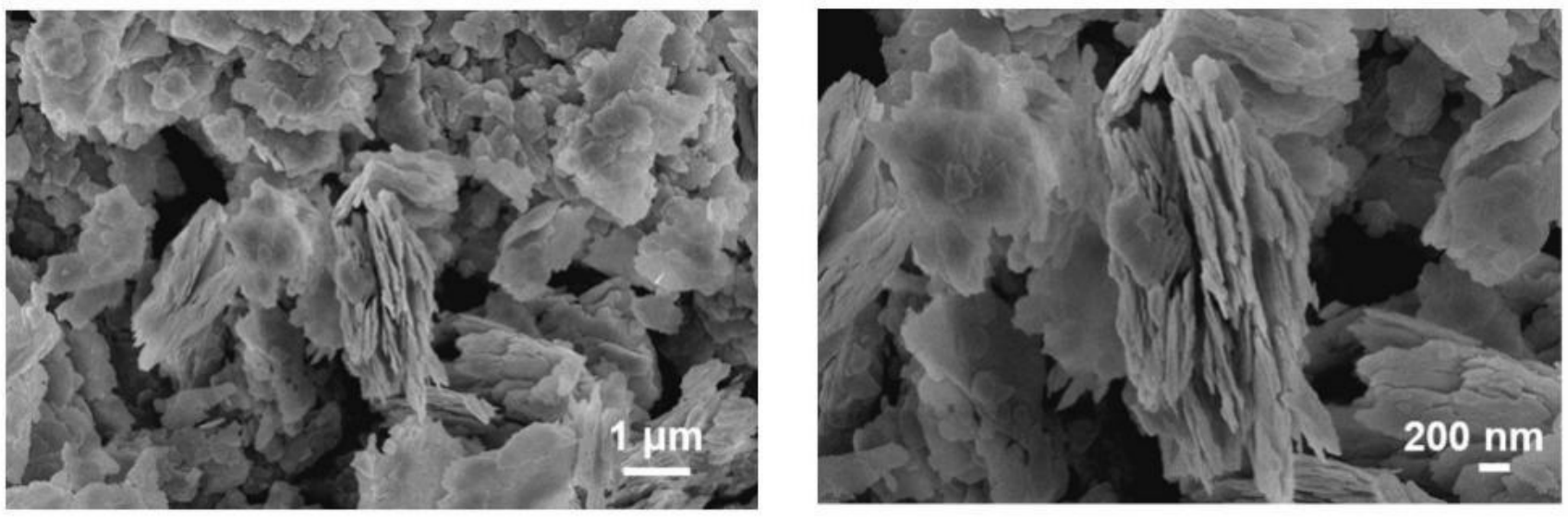

Figure S6. SEM image of the $Ni_{10}Nd_1$ catalyst.

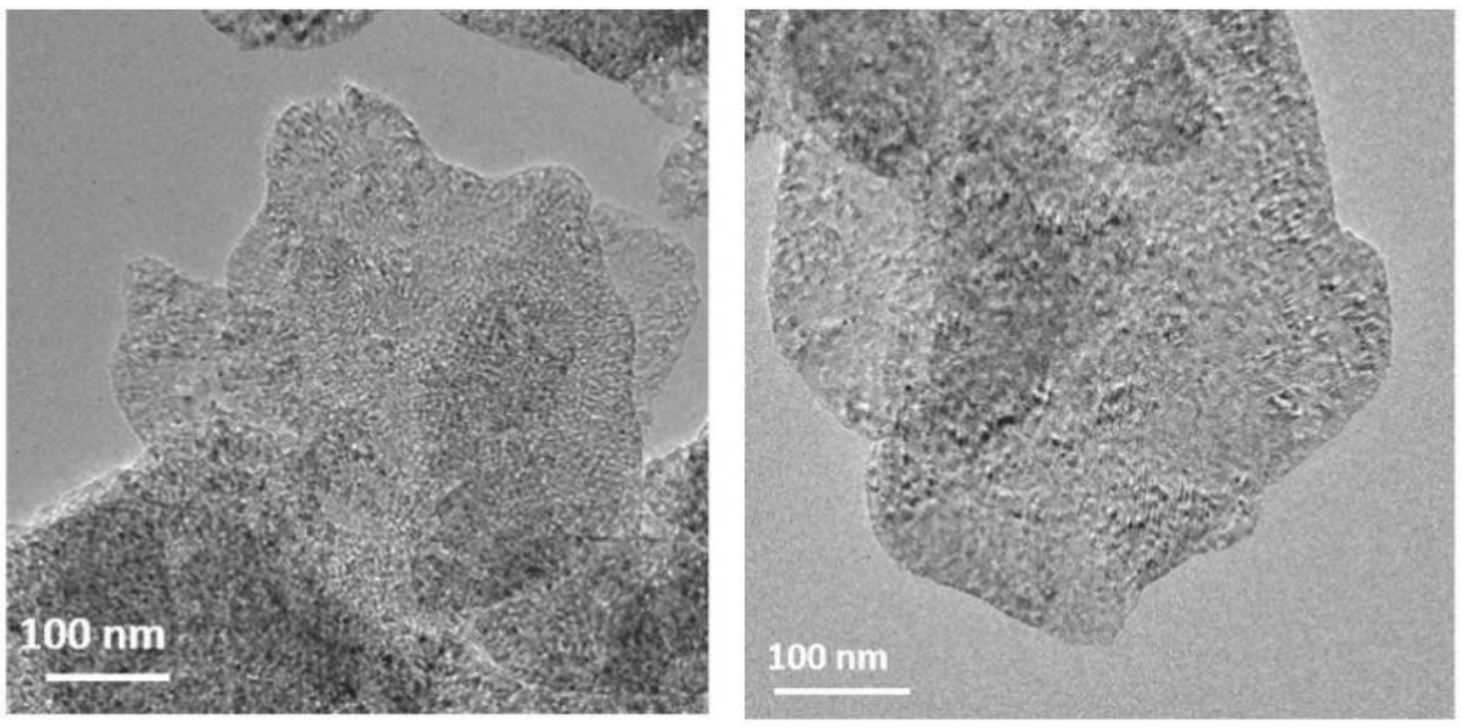

Figure S7. TEM image of the Ni_5Nd_1 catalyst.

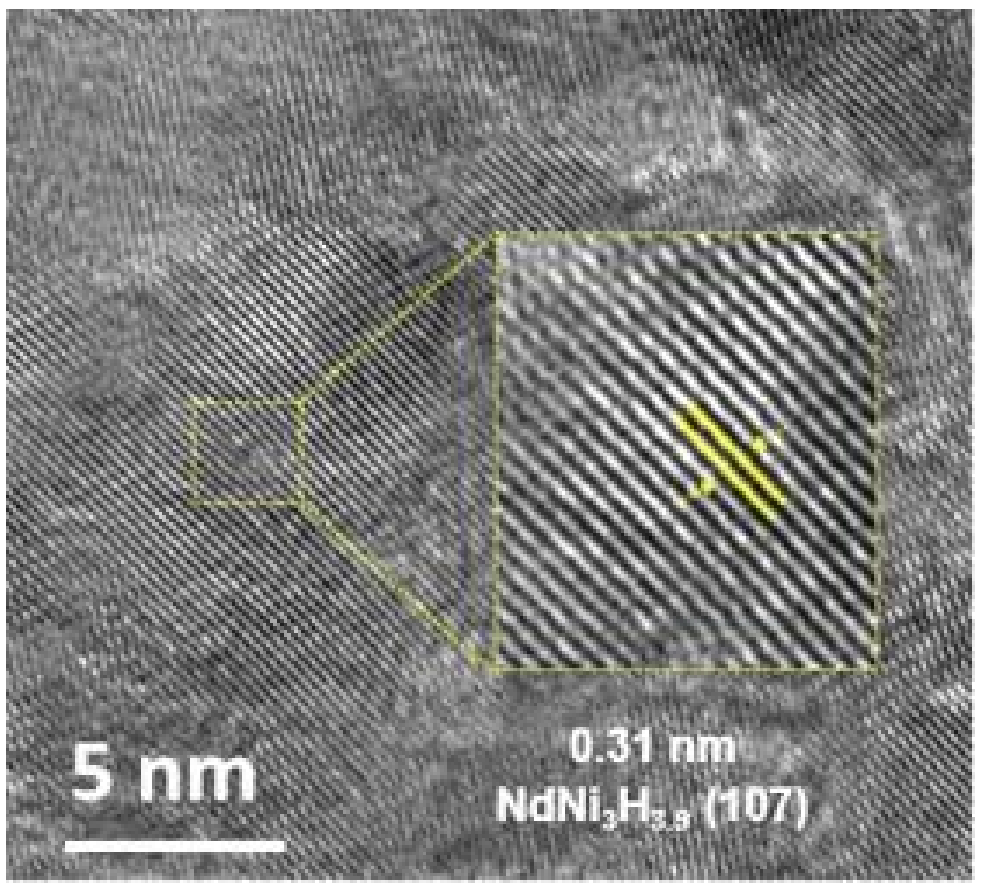

Figure S8. HRTEM image of the Ni_5Nd_1 catalyst.

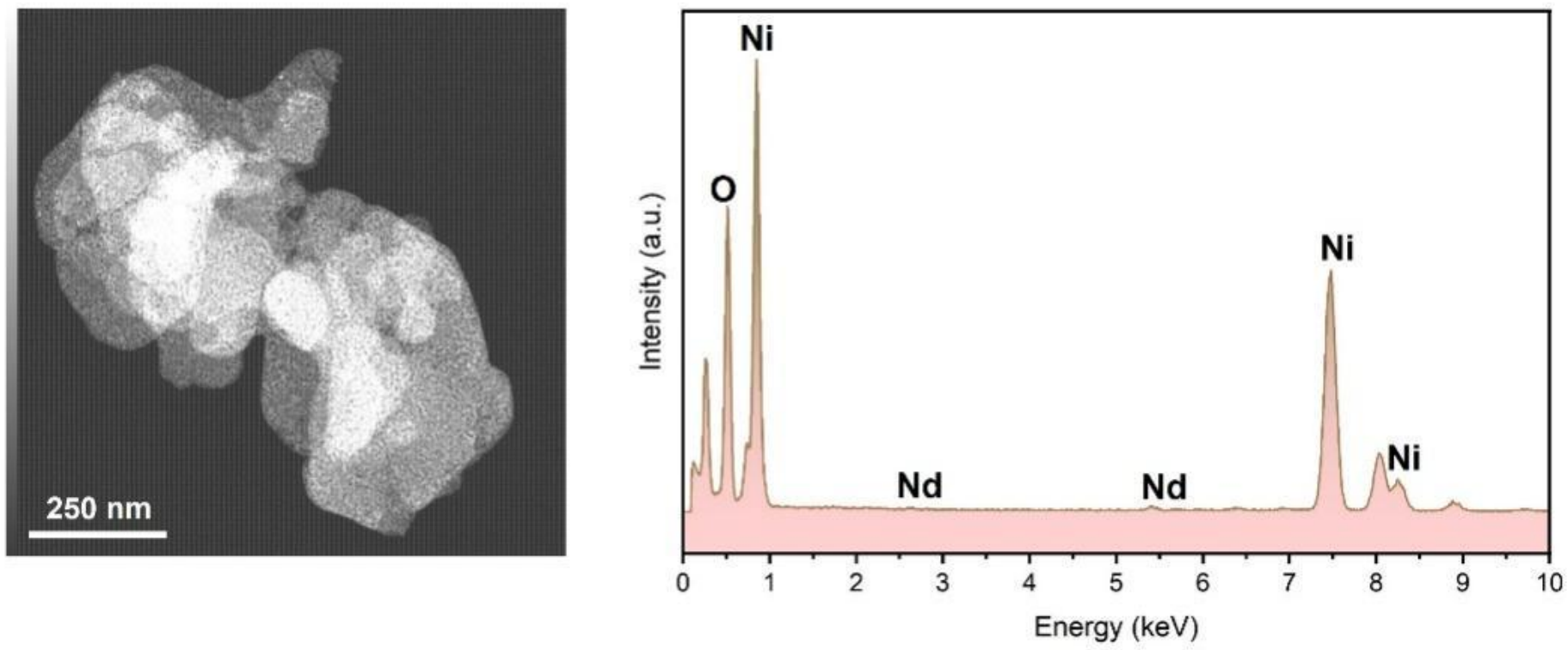

Figure S9. HADDF image and the corresponding EDS plot of the Ni_5Nd_1 catalyst.

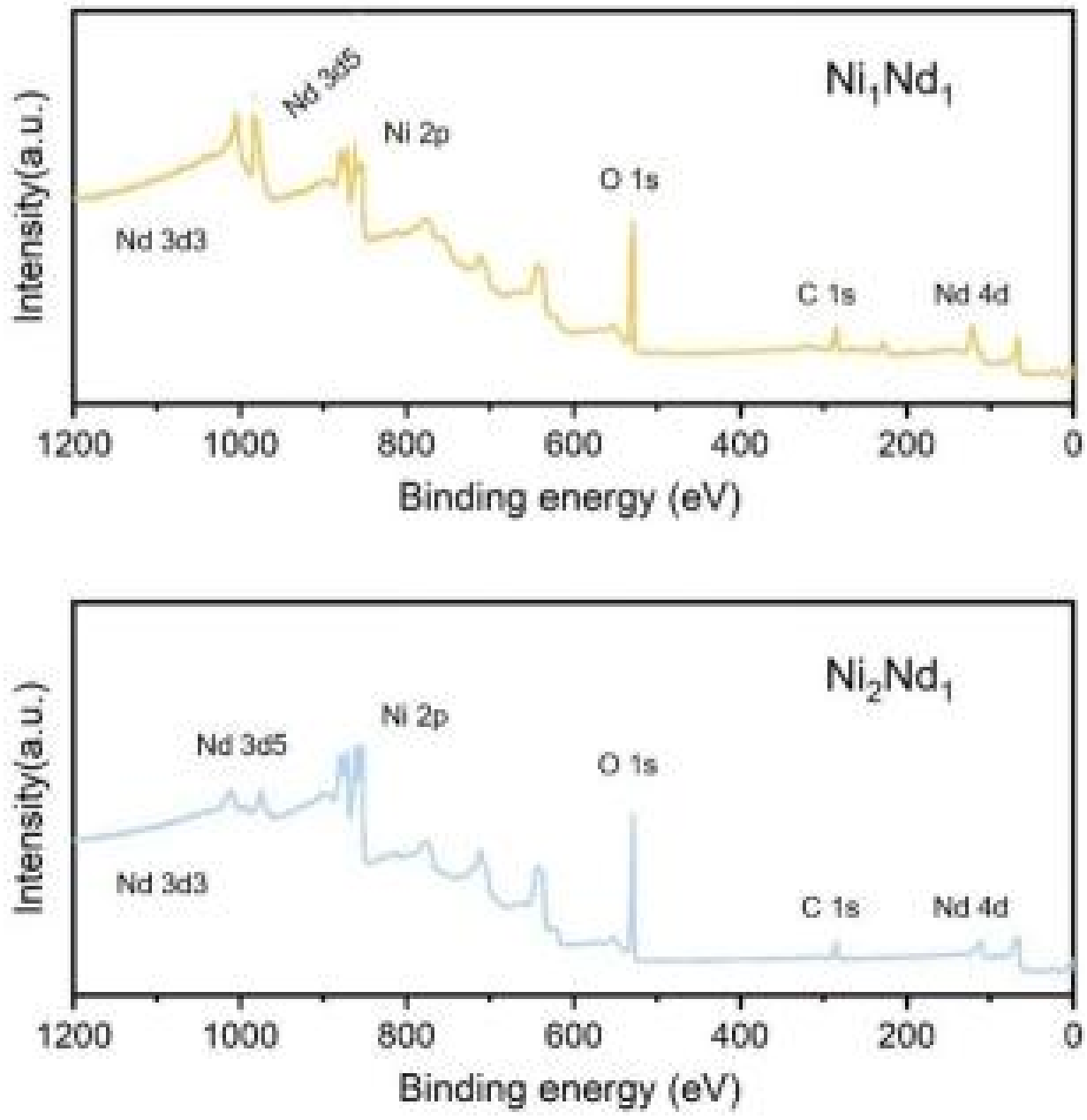

Figure S10. XPS survey spectra of the Ni_1Nd_1 and Ni_2Nd_1 catalyst.

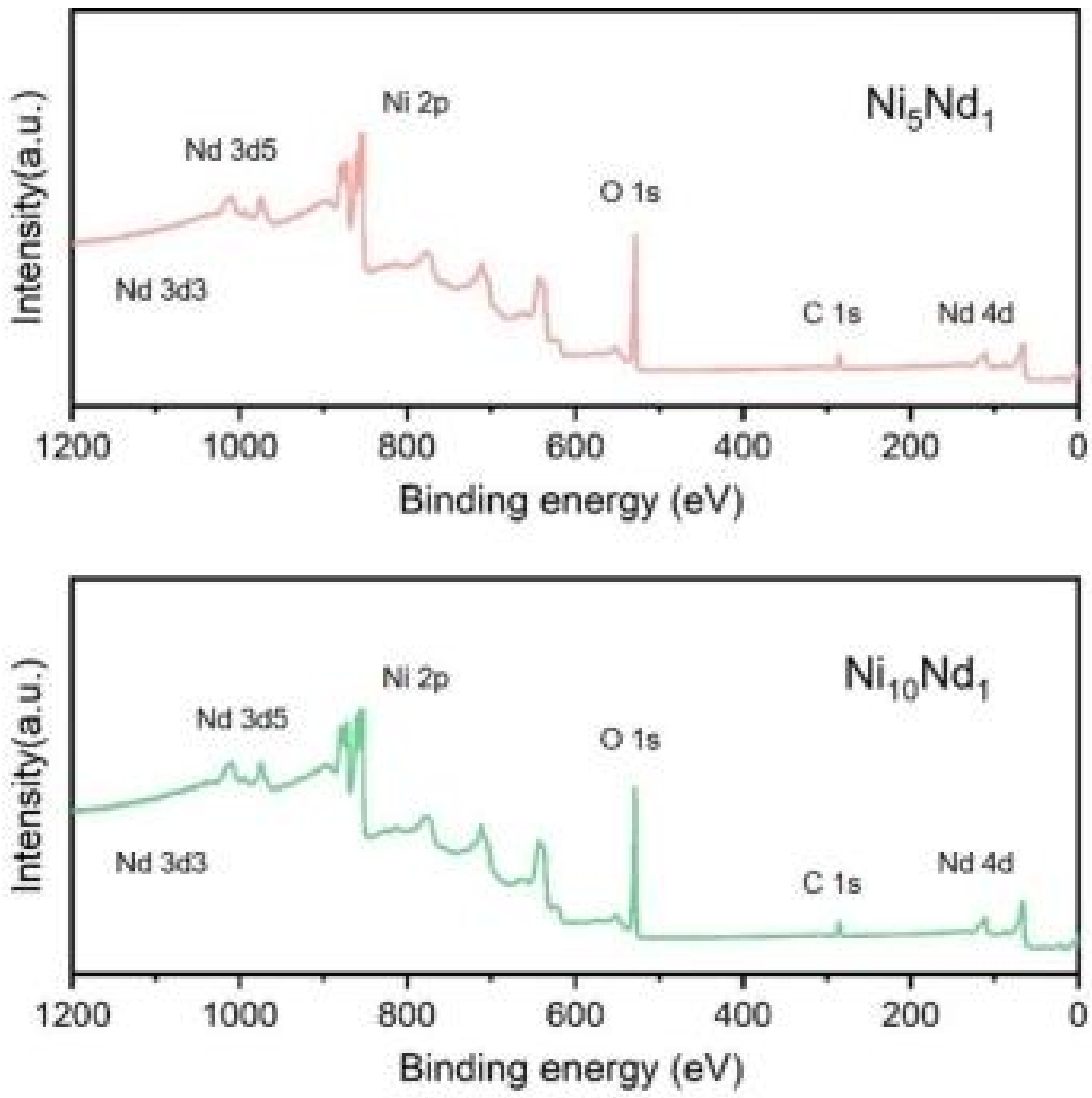

Figure S11. XPS survey spectra of the Ni_5Nd_1 and $Ni_{10}Nd_1$ catalyst.

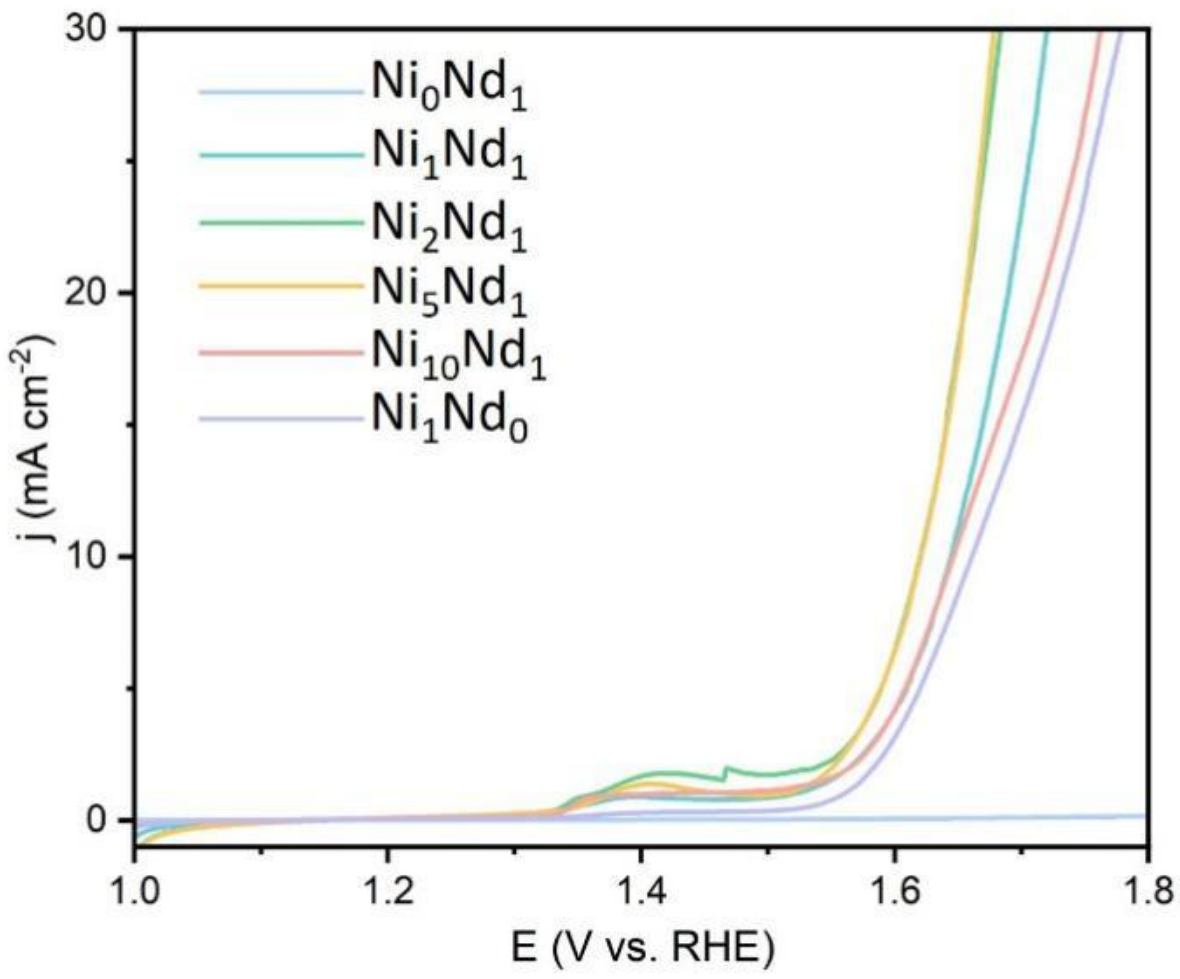

Figure S12. LSV plots of the Nd-Ni oxides catalysts for OER.

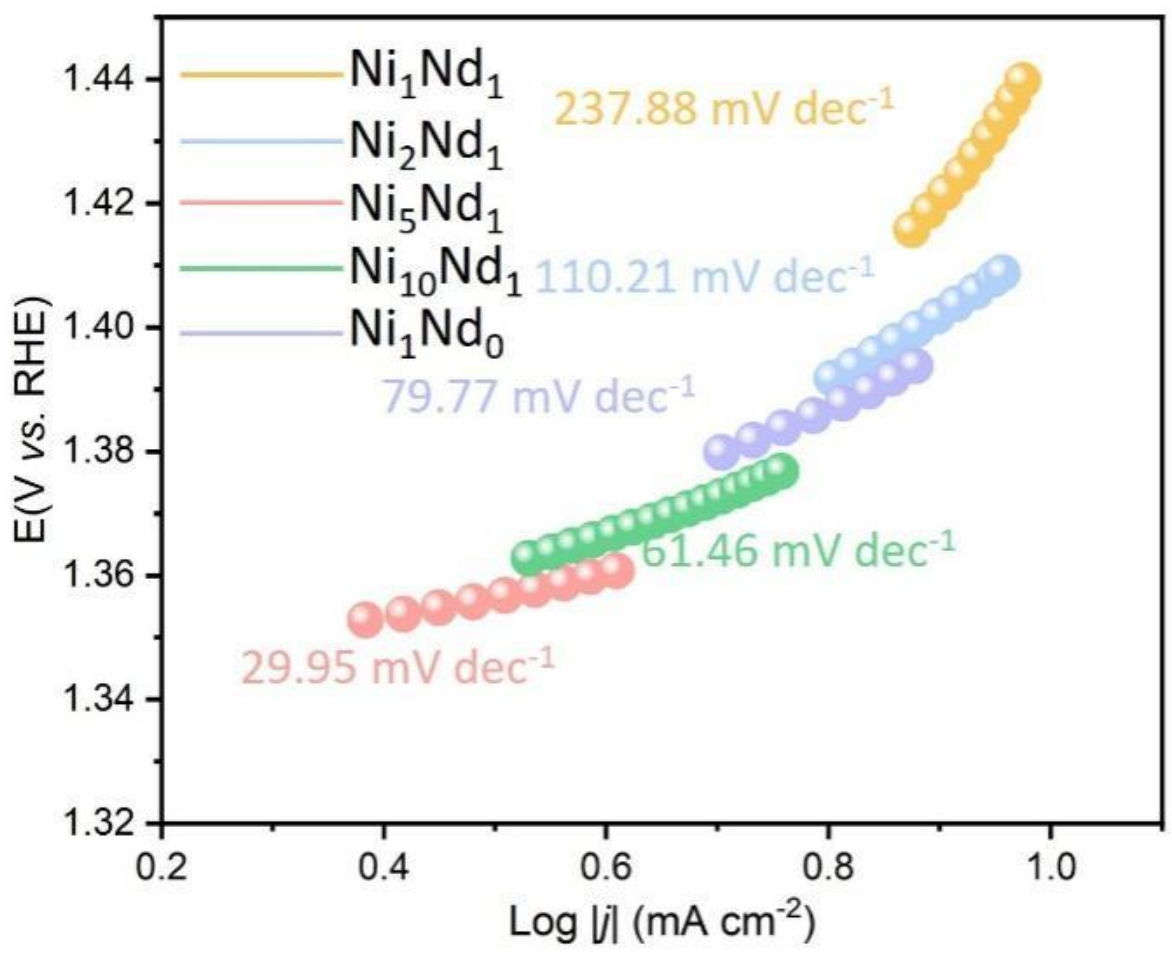

Figure S13. Tafel plots of the Nd-Ni oxides catalysts for UOR.

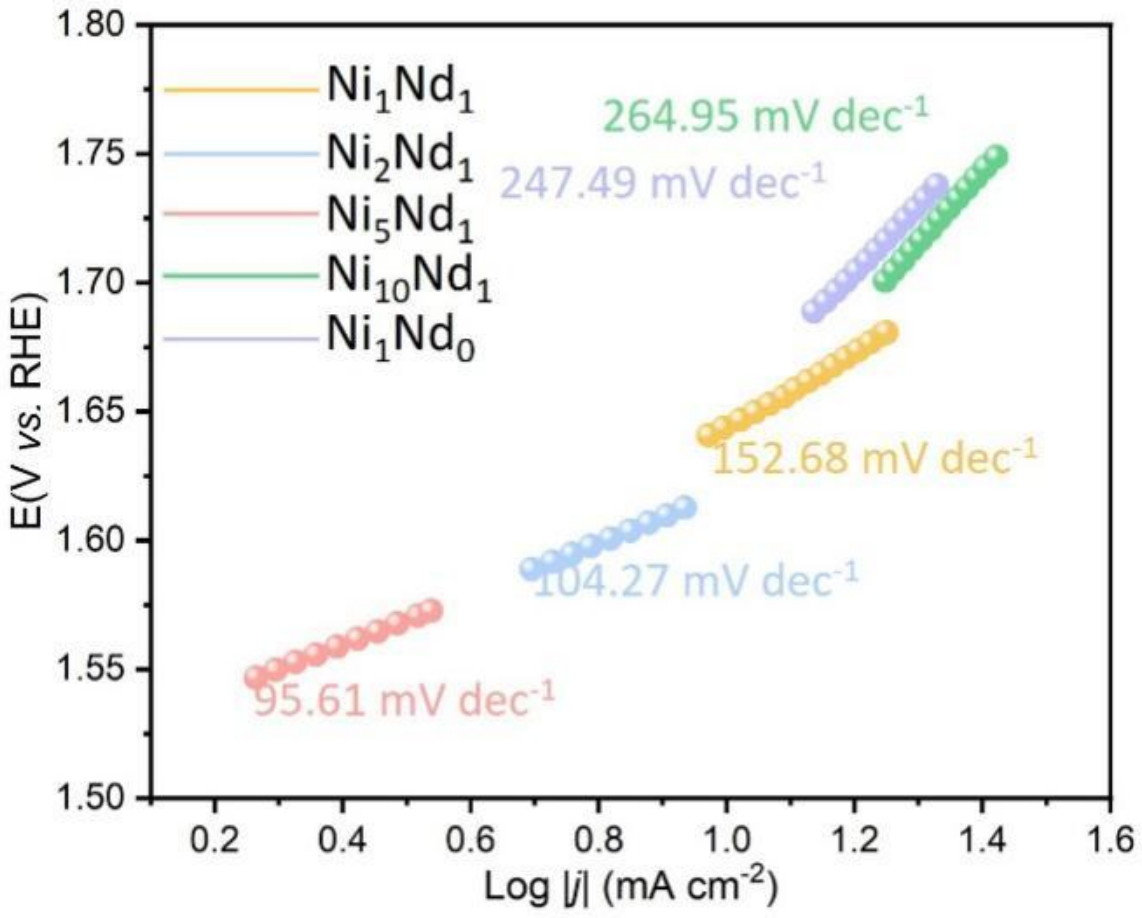

Figure S14. Tafel plots of the Nd-Ni oxides catalysts for OER.

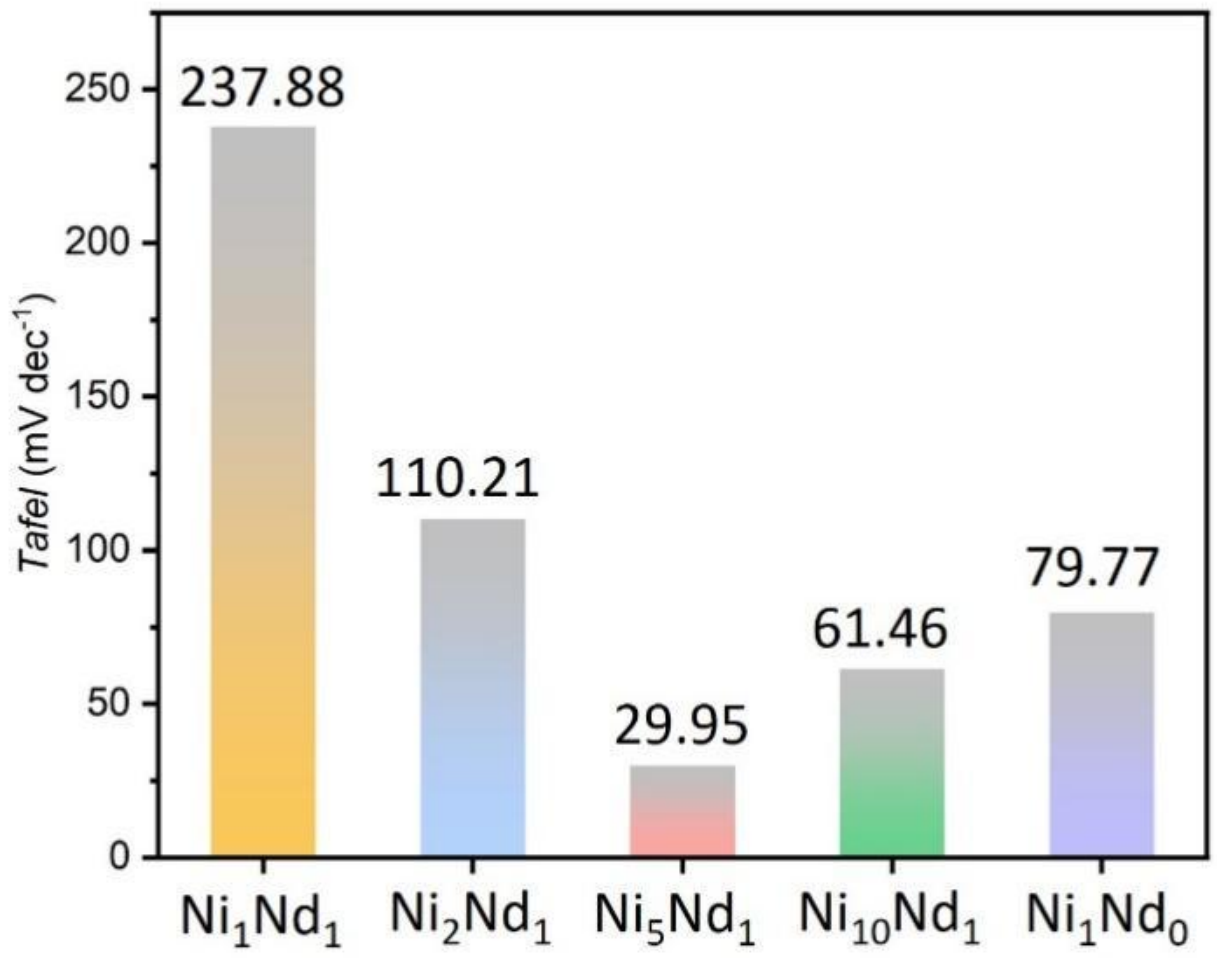

Figure S15. Comparison on Tafel slopes of the Nd-Ni oxides catalysts for UOR.

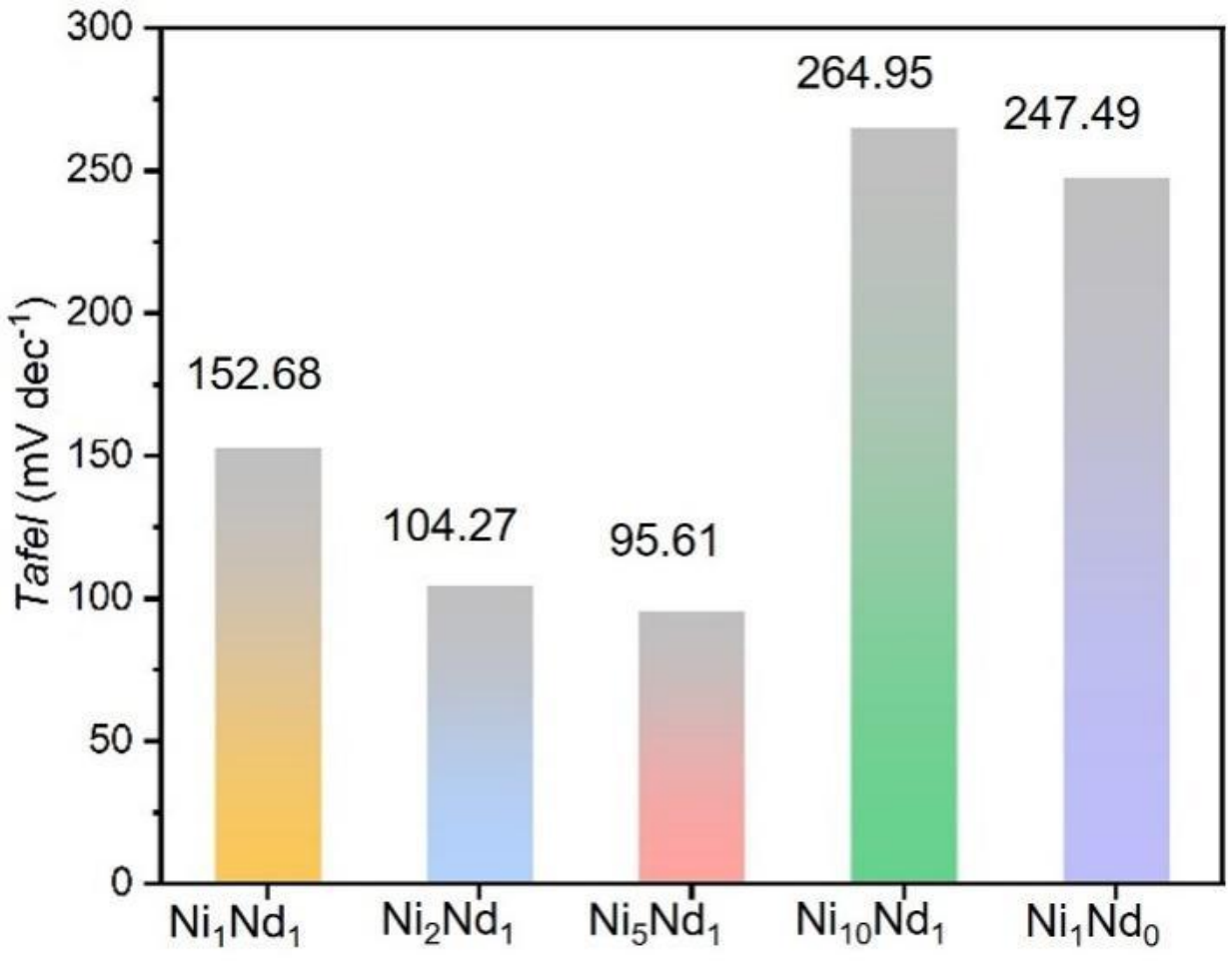

Figure S16. Comparison on Tafel slopes of the Nd-Ni oxides catalysts for OER.

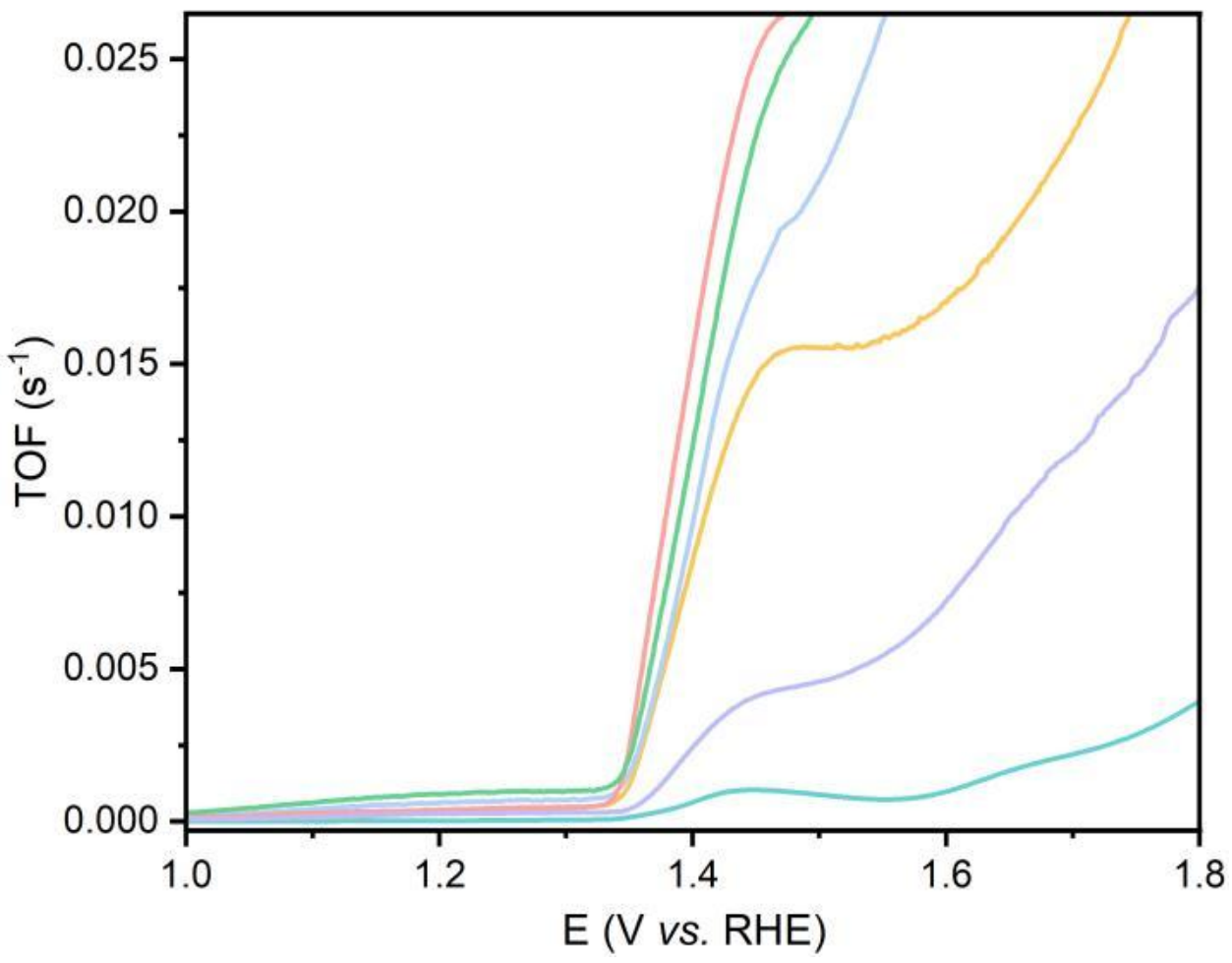

Figure S17. TOF plots of the Nd-Ni oxides catalysts for UOR.

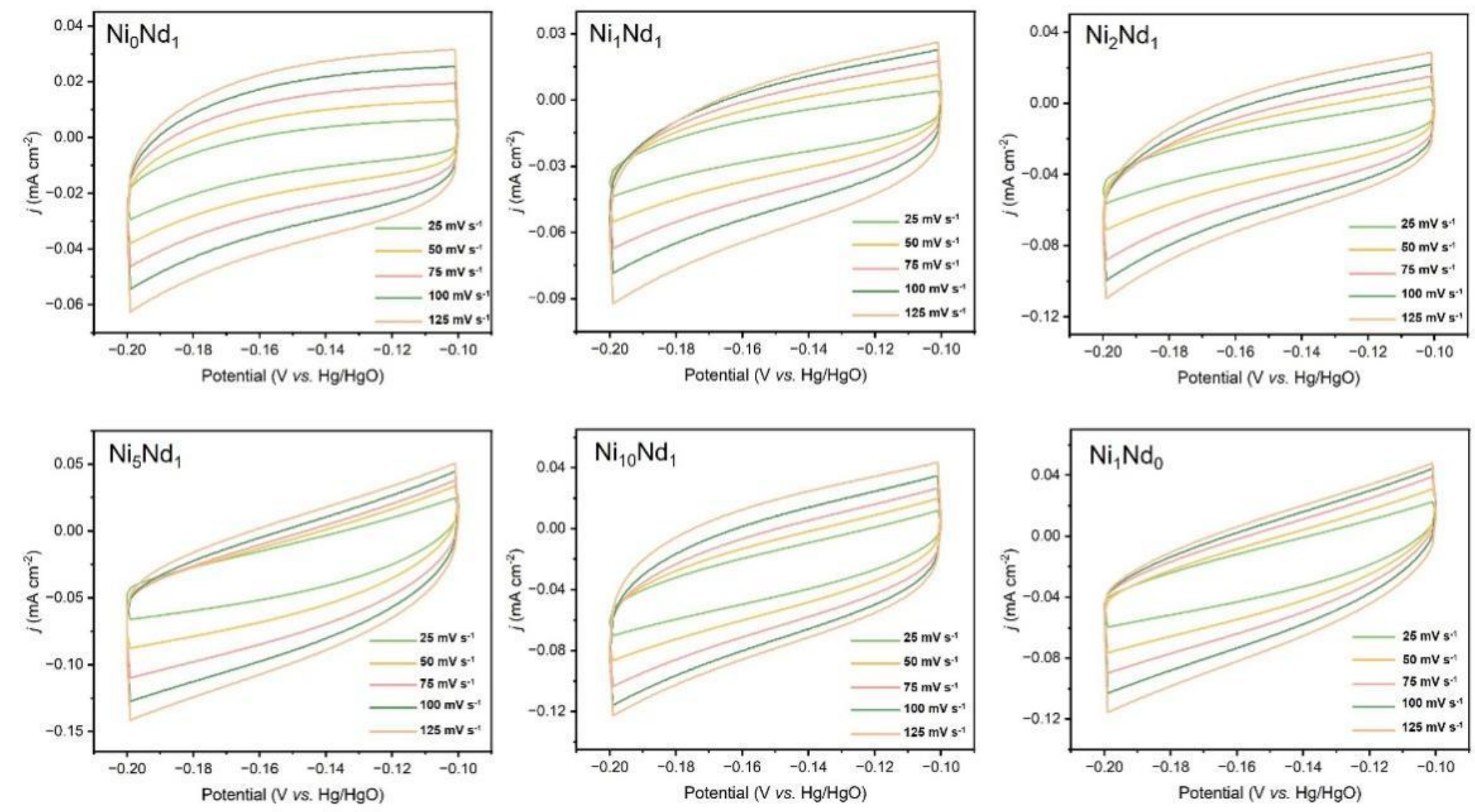

Figure S18. CV curves at different scan rates of the Nd-Ni oxides catalysts for UOR.

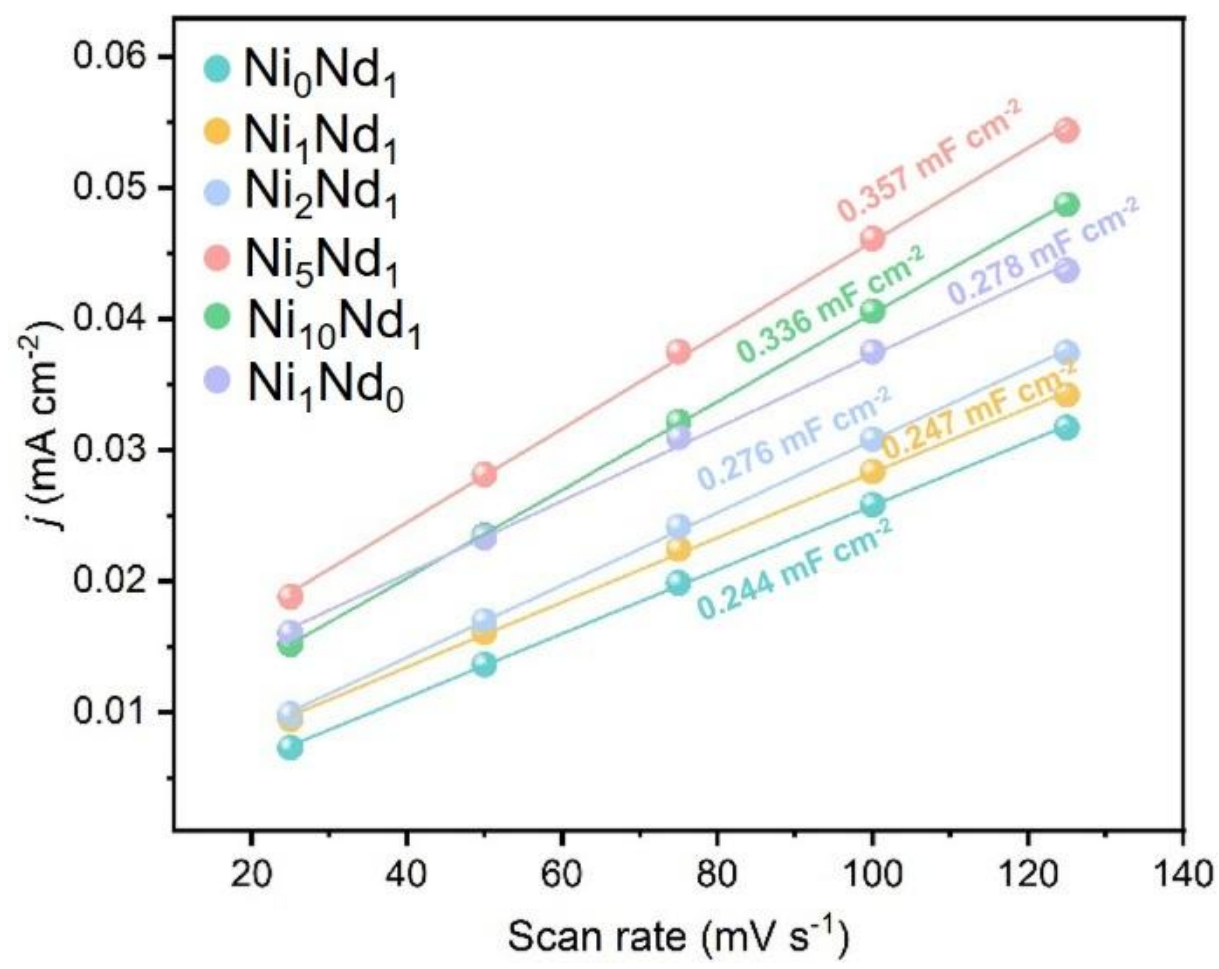

Figure S19. Linear curves of the Nd-Ni oxides catalysts for UOR.

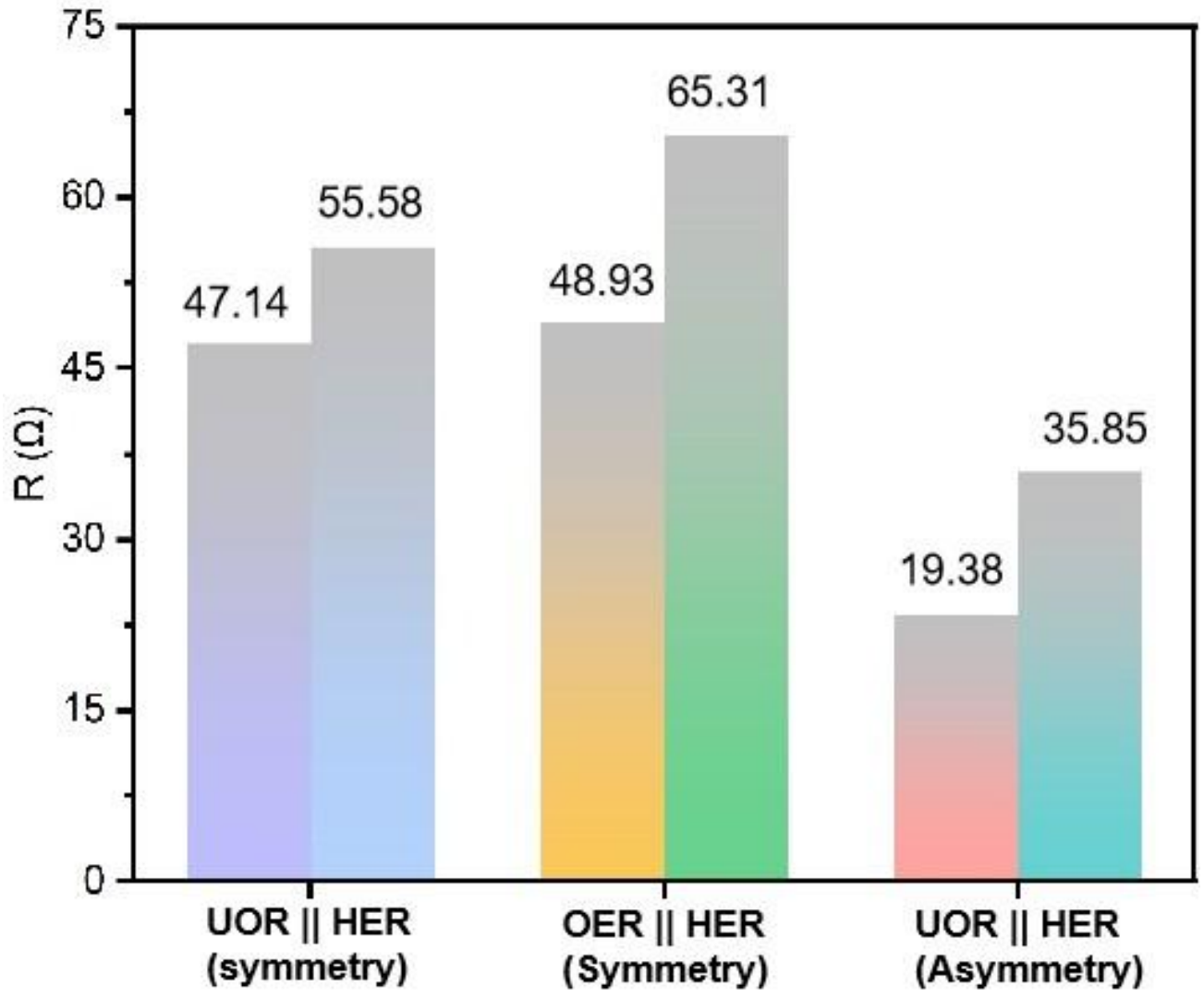

Figure S20. EIS R_{ct} values of two-electrode systems before and after UOR.

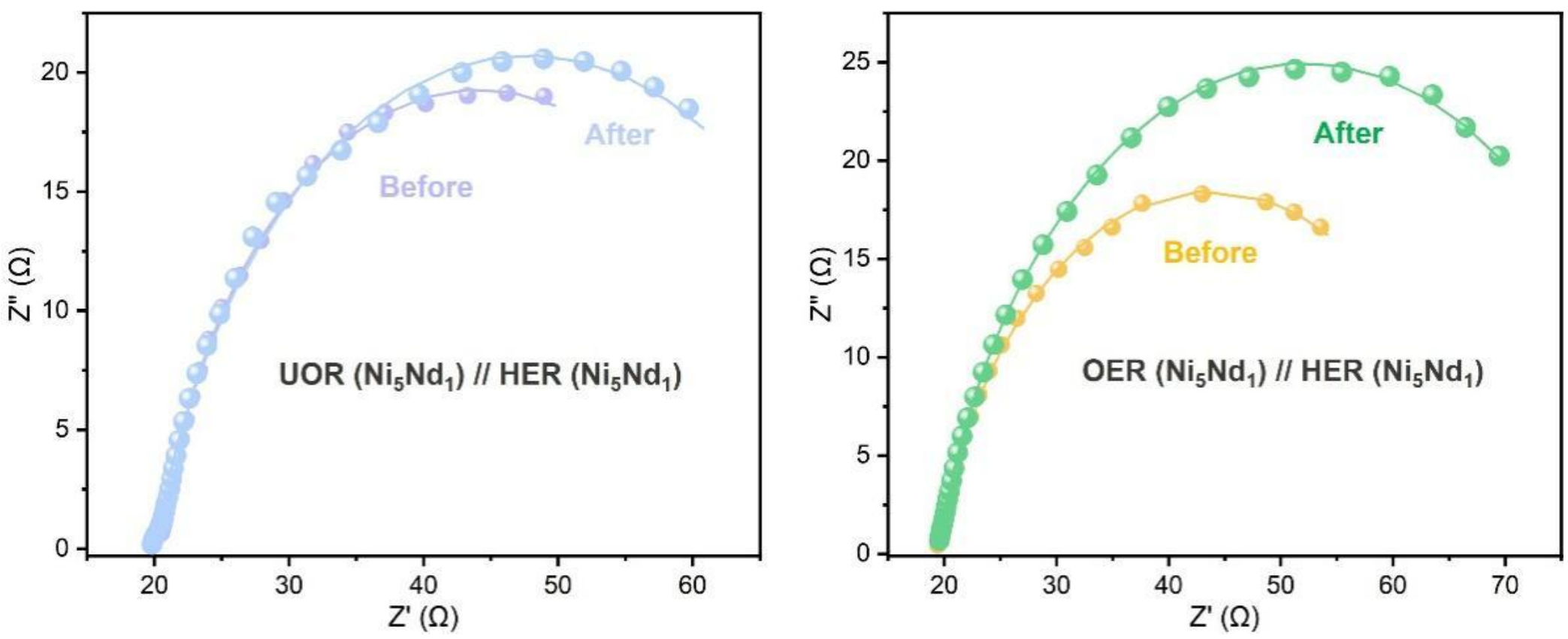

Figure S21. EIS curves of the Ni_5Nd_1@CC catalyst based two-electrode symmetrical cells before and after UOR/HER and OER/HER.

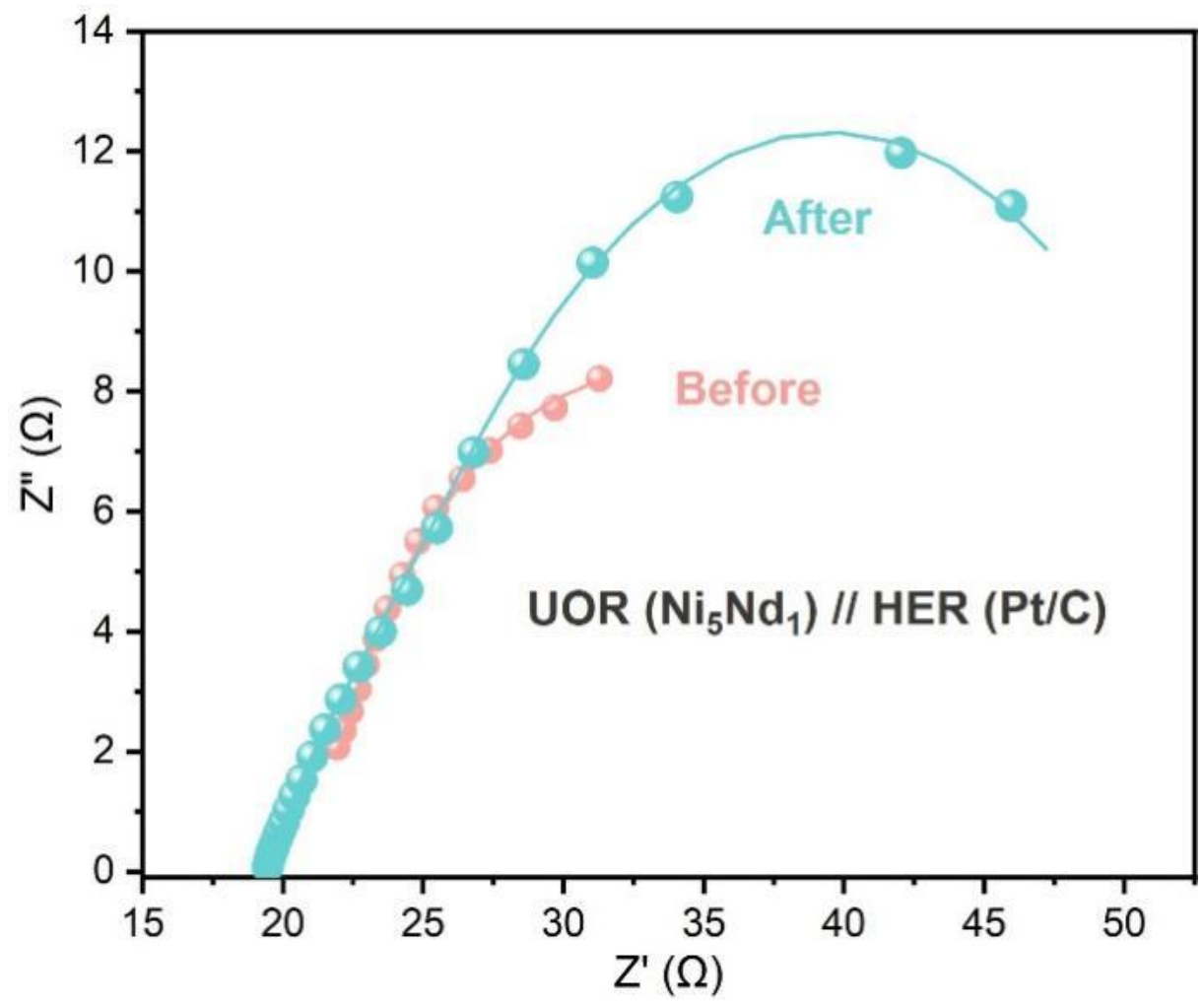

Figure S22. EIS curves of the Ni_5Nd_1@CC catalyst based two-electrode asymmetrical cell before and after UOR/HER.

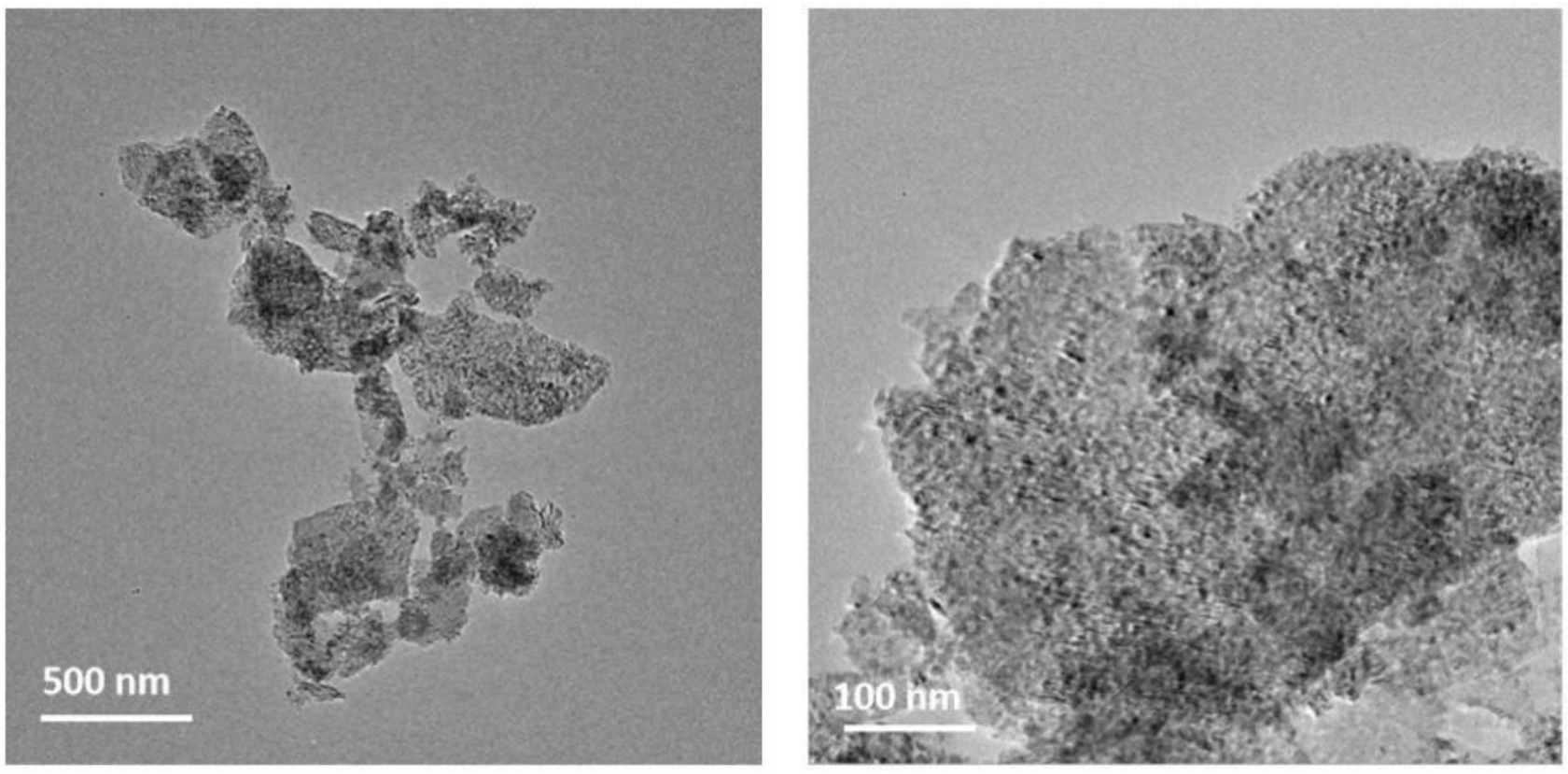

Figure S23. TEM image of the Ni_5Nd_1 catalyst for UOR after long-term operation.

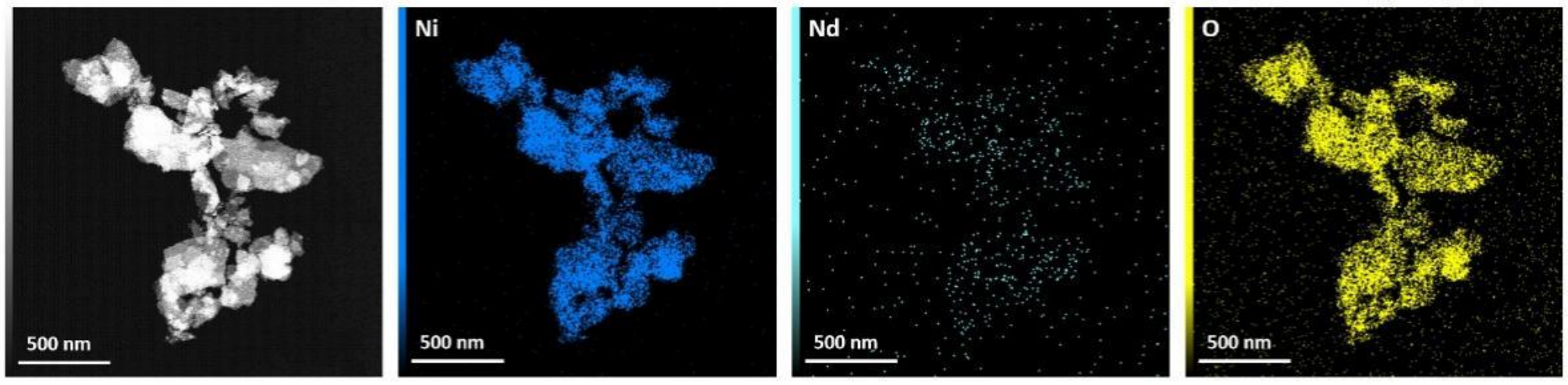

Figure S24. HADDF image and the corresponding element mapping patterns of the Ni_5Nd_1 catalyst for UOR after long-term operation.

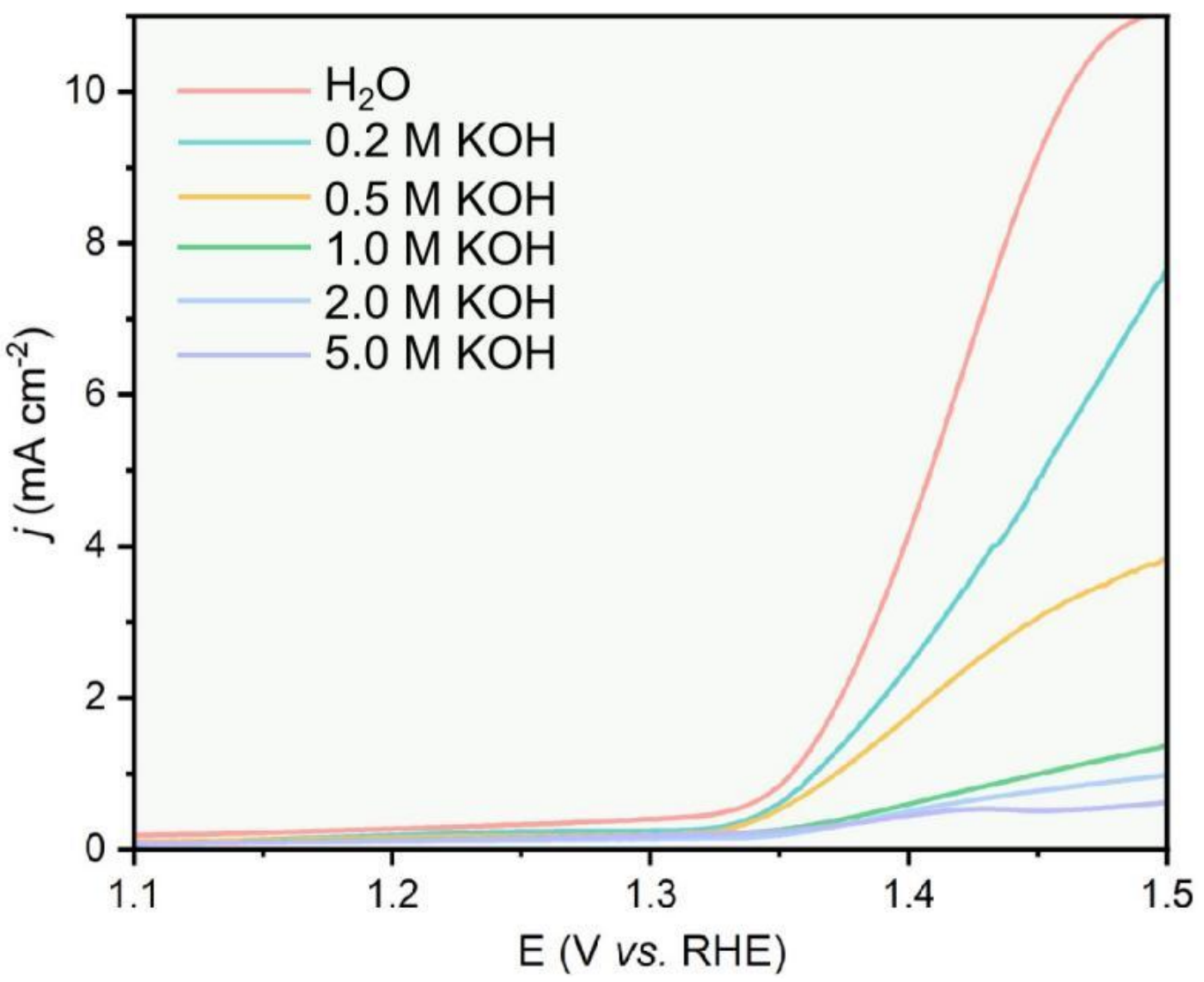

Figure S25. LSV plots of the Ni_5Nd_1 catalyst treated in different concentration KOH solutions.

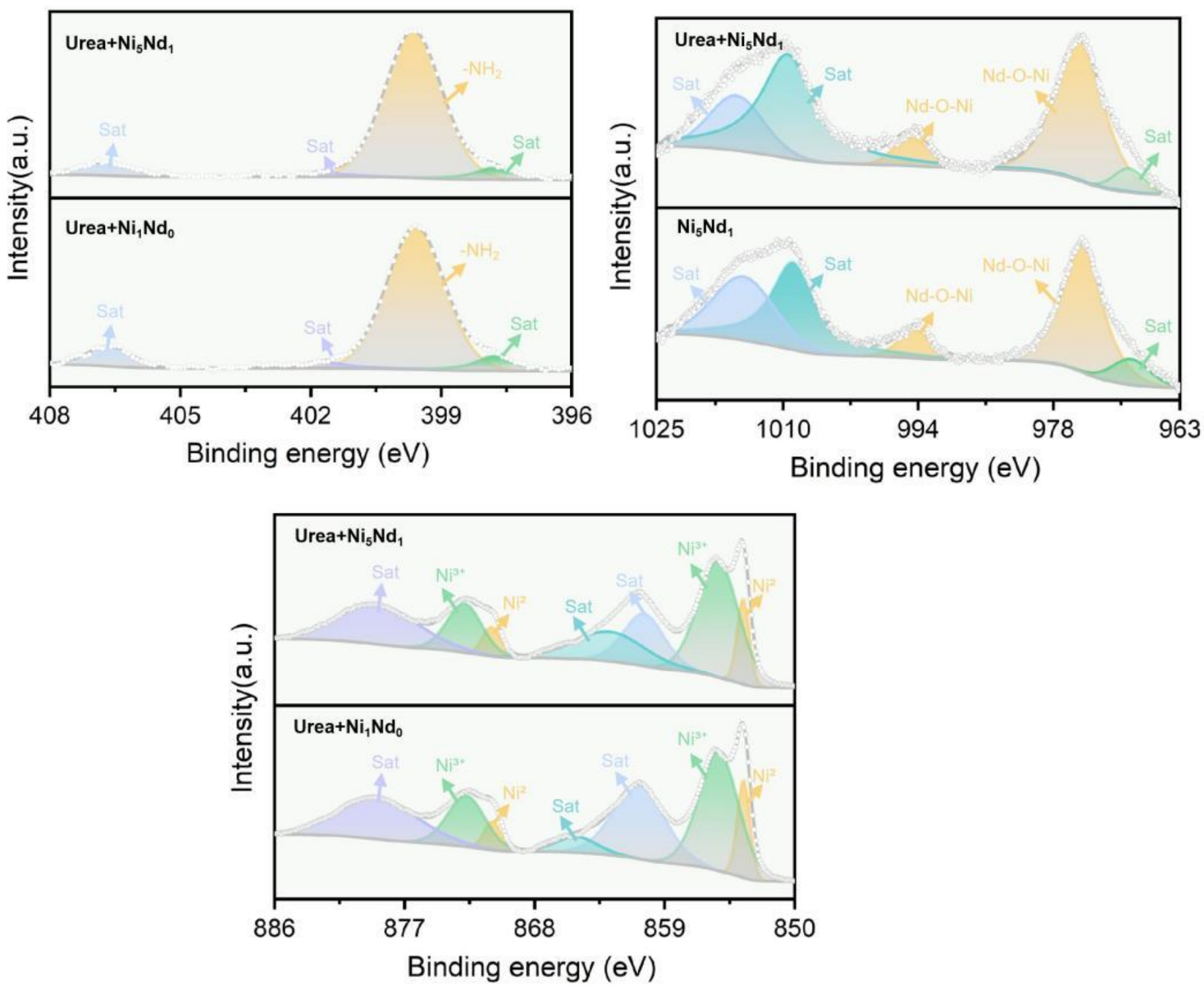

Figure S26. XPS spectra on the catalyst with and without urea adsorption.

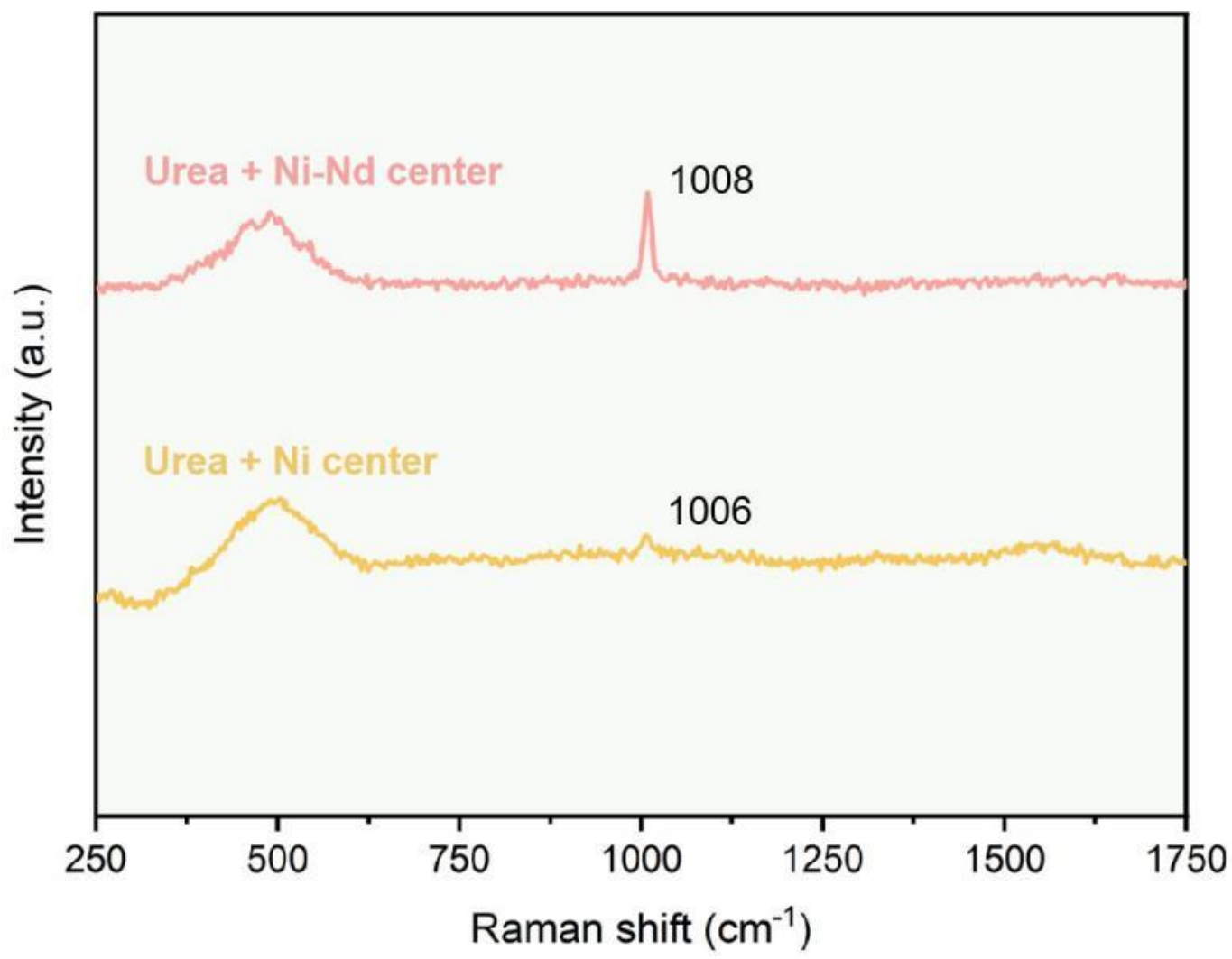

Figure S27. Raman spectra on the catalyst with and without urea adsorption.

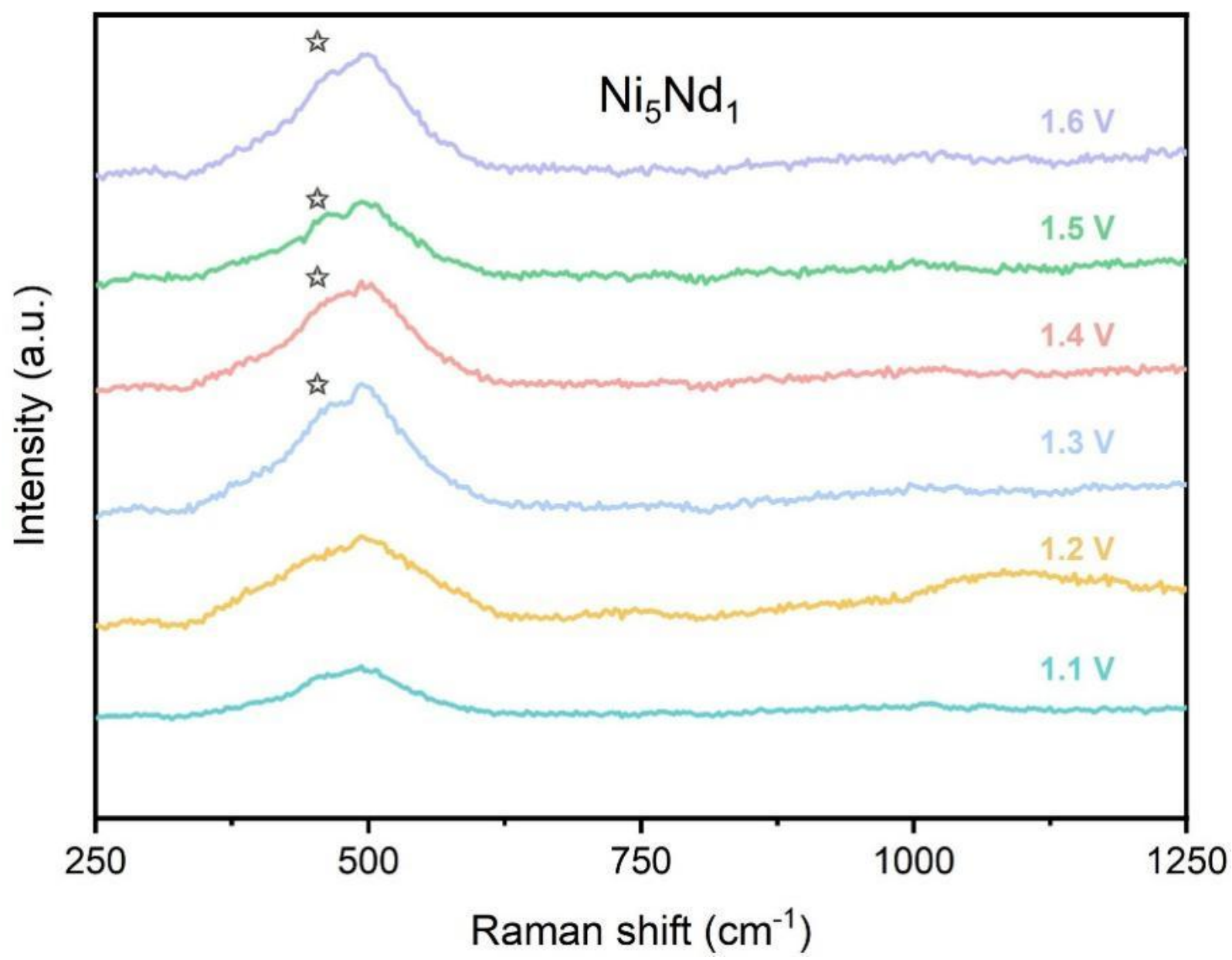

Figure S28. Potential-dependent Raman spectra of the Ni_5Nd_1 catalyst for alkaline UOR.

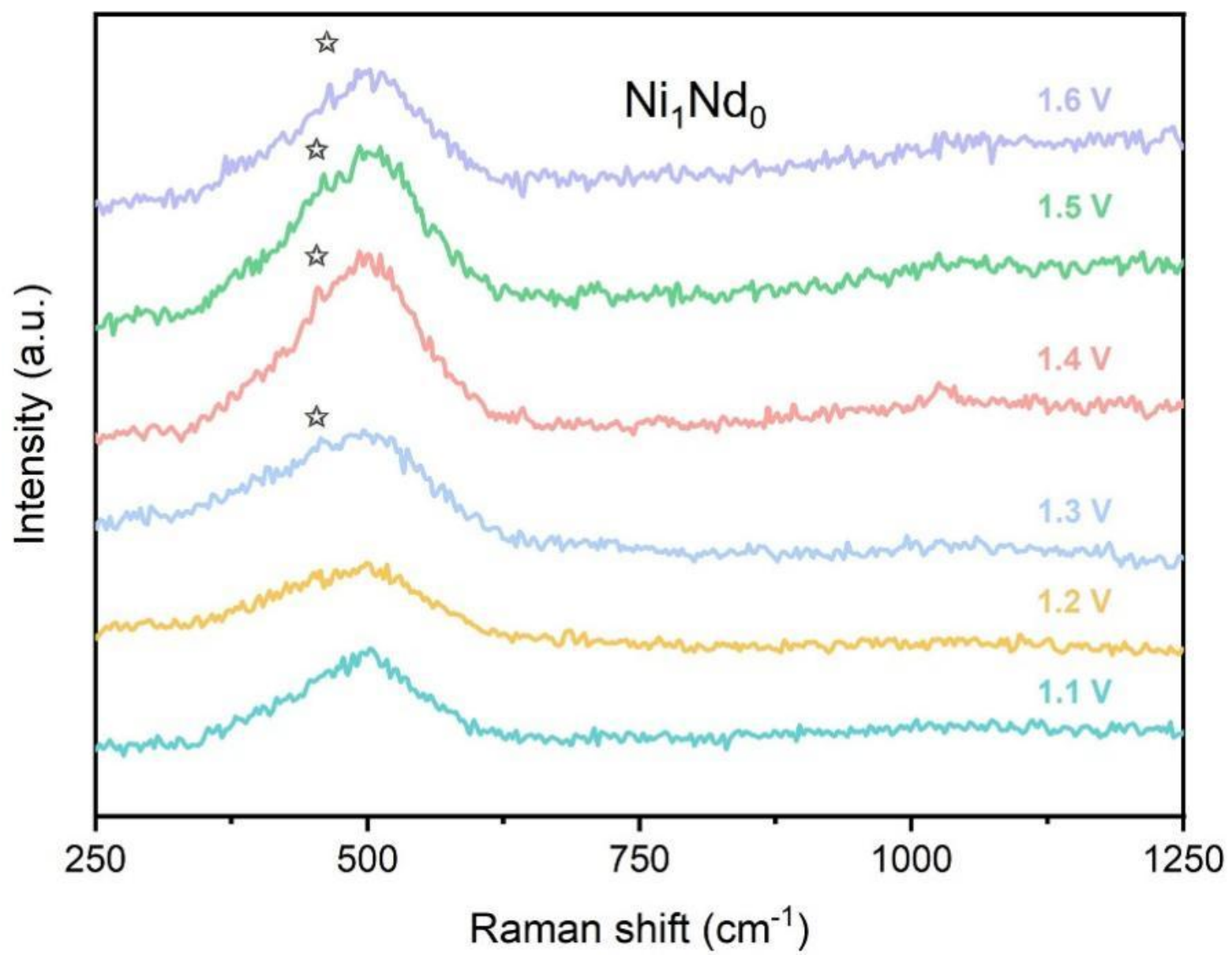

Figure S29. Potential-dependent Raman spectra of the Ni_1Nd_0 catalyst for alkaline UOR.

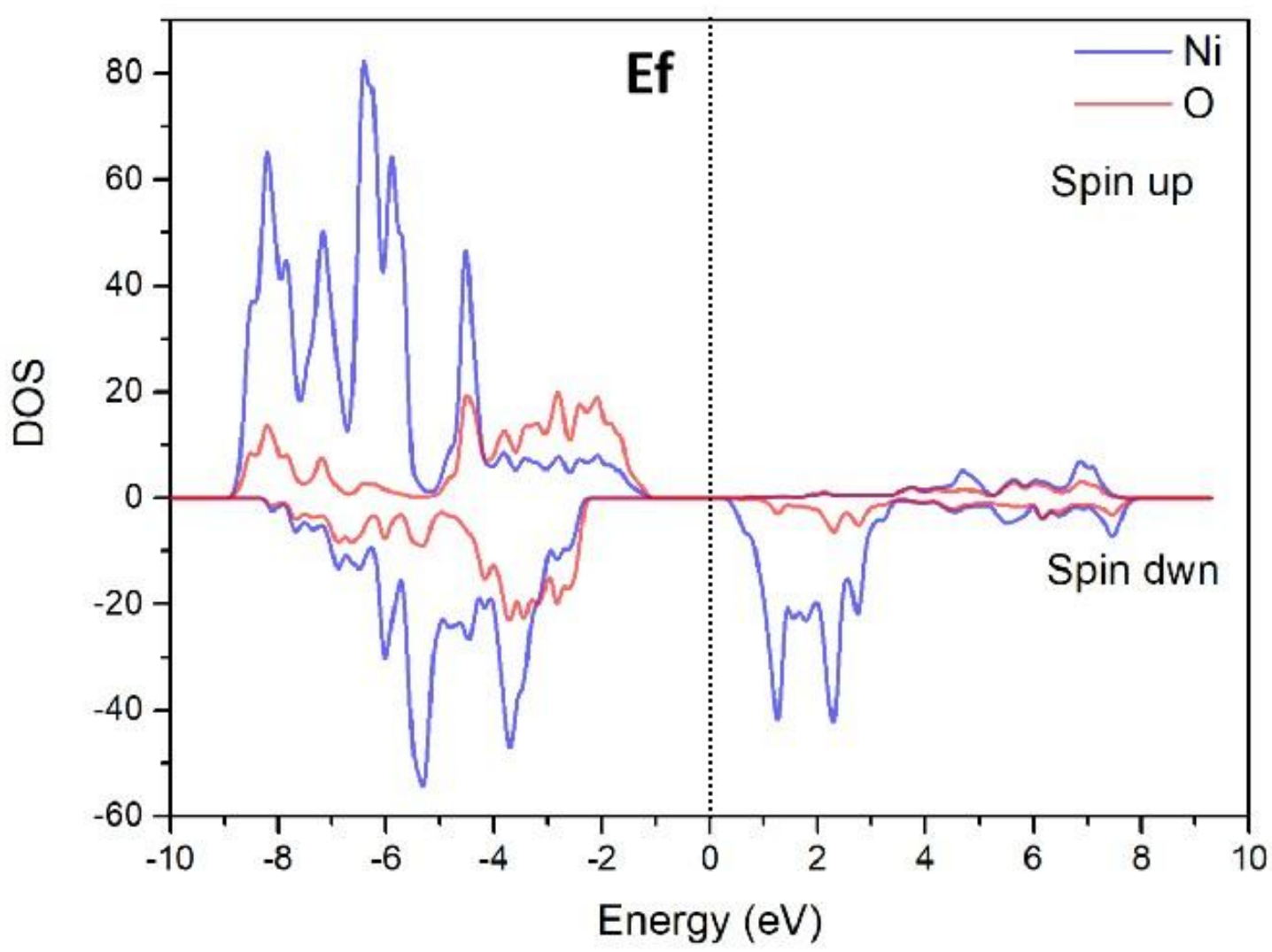

Figure S30. DOS plots of the Ni-O model.

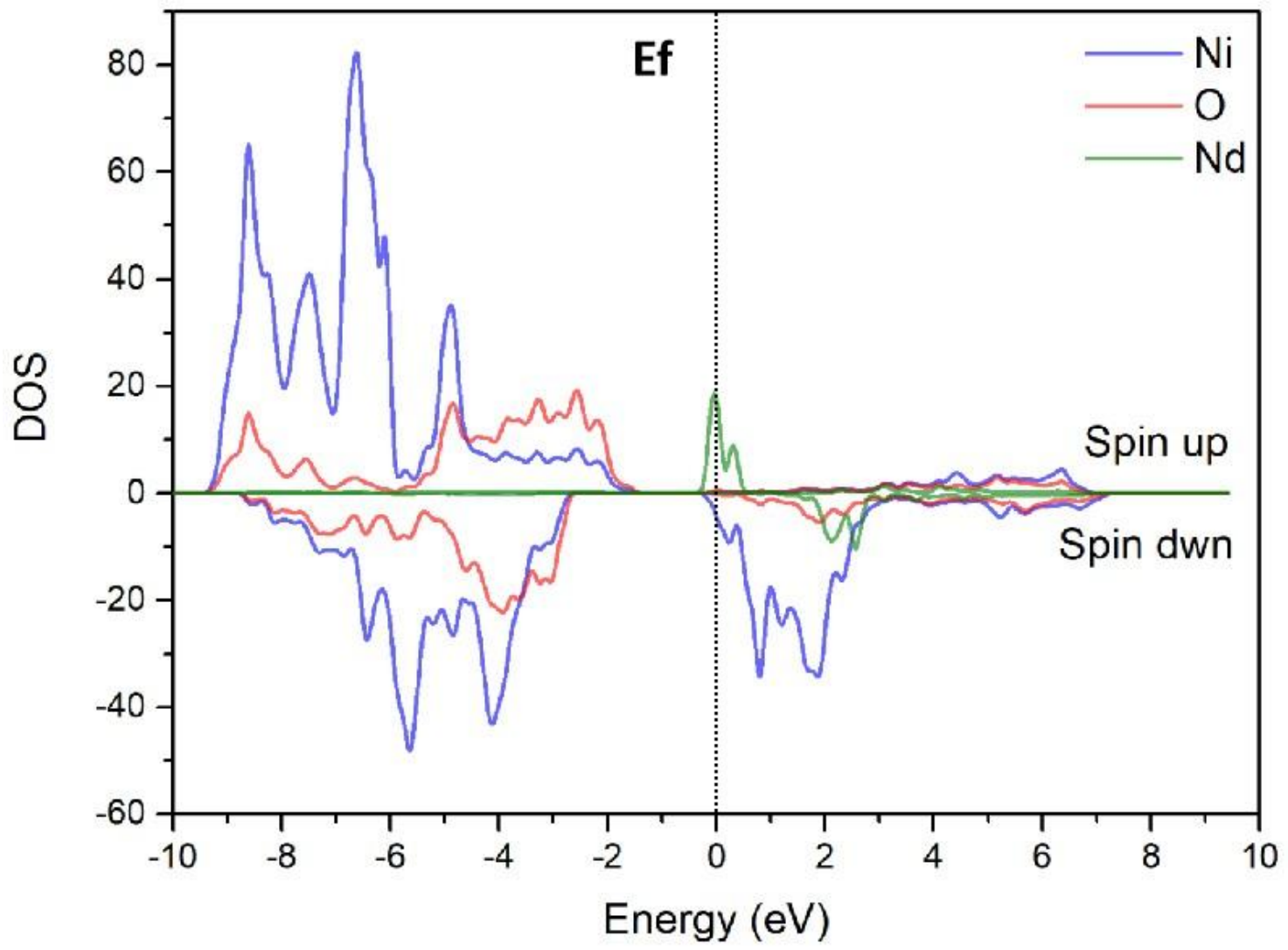

Figure S31. DOS plots of the Nd-Ni-O model.

Table S1. Chemical composition of different Nd-Ni oxide catalysts based on ICP-OES tests

Catalysts	Ni (Wt%)	Nd (Wt%)
**Ni_1Nd_1**	28.32	38.81
**Ni_2Nd_1**	74.14	2.19
**Ni_5Nd_1**	75.26	0.60
**$Ni_{10}Nd_1$**	77.42	0.06

Table S2. Comparison of UOR activity of Ni_5Nd_1@CC with reported catalysts.

Catalysts	Electrolyte	Potential (j_{10}, V)	Tafel slope (mV dec^{-1})	Ref
**Ni_5Nd_1@CC**	1.0 M KOH + 0.33 M Urea	1.347	37.92	This work
**O_{vac}-V_1-$Ni(OH)_2$**	1.0 M KOH + 0.33 M Urea	≈ 1.38	43.87	[1]
**NNO_2NO_8/GCE**	1.0 M KOH + 0.80 M Urea	1.35	41.7	[2]
**Ni SAs-NC**	1.0 M KOH + 0.30 M Urea	1.39	42	[3]
**$Ni_2P/Ni_{0.96}S$**	1.0 M KOH + 0.50 M Urea	≈ 1.36	56	[4]
**β-$NiMoO_4$**	1.0 M KOH + 0.50 M Urea	1.38	40	[5]
**$(Ni_{0.25}Fe_{0.75})_3S_2$/NF**	1.0 M KOH + 0.33 M Urea	1.38	87.8	[6]
**$Co_2P_4O_{12}$/CC**	1.0 M KOH + 0.33 M Urea	1.39	69	[7]
**NiFe LDH**	1.0 M KOH + 0.33 M Urea	1.42	47	[8]
**Ni-MOF-0.5**	1.0 M KOH + 0.50 M Urea	1.381	52	[9]
**NiO/Ni@C**	1.0 M KOH + 0.33 M Urea	1.35	98	[10]
**Ni_3N/NF**	1.0 M KOH + 0.50 M Urea	≈ 1.35	41	[11]
**NiS/NiS_2-Ni@NCNT-50**	1.0 M KOH + 0.50 M Urea	1.37	39	[12]
**NiCr LDH**	1.0 M KOH + 0.50 M Urea	1.361	38.3	[13]
**$CoMn_2O_4$**	1.0 M KOH + 0.50 M Urea	≈ 1.37	68	[14]
**NiFe-LDH@NiMoP/NF**	1.0 M KOH + 0.50 M Urea	1.35	84.9	[15]
**V-Ni_3N/NF**	1.0 M KOH + 0.50 M Urea	1.361	45	[16]

**$NiMoS_2$/NF**	1.0 M KOH + 0.33 M Urea	1.39	64	[17]
**NiS@Ni_3S_2**	1.0 M KOH + 0.50 M Urea	1.37	40	[18]
**Ni@NCNT**	1.0 M KOH + 0.50 M Urea	1.39	76.3	[19]
**Ni@S-C-500**	1.0 M KOH + 0.33 M Urea	1.41	98.9	[20]
**$NiSe_2$/$CoSe_2$**	1.0 M KOH + 0.33 M Urea	1.39	39	[21]
**Ni/MNO-10**	1.0 M KOH + 0.50 M Urea	1.37	51.2	[22]
**Fe-Ni_3S_2@$FeNi_3$-8**	1.0 M KOH + 0.33 M Urea	1.40	69	[23]
**Ni_2P/Fe_2P/NF**	1.0 M KOH + 0.50 M Urea	1.36	79.1	[24]
**V_8C_7/CoP-0.11**	1.0 M KOH + 0.33 M Urea	1.40	189	[25]
**Ni-V_2O_3/NF**	1.0 M KOH + 0.50 M Urea	1.35	60.48	[26]
**GN-2**	1.0 M KOH + 0.33 M Urea	1.38	44	[27]
**NiFe-LDH@rGO**	1.0 M KOH + 0.33 M Urea	1.45	69	[28]
**H-NiFe-LDH/NF**	1.0 M KOH + 0.33 M Urea	1.397	41.7	[29]
**Ni_3S_2-CDs@NF**	1.0 M KOH + 0.30 M Urea	1.35	96	[30]
**CrCoNiFe**	1.0 M KOH + 0.33 M Urea	1.561	61	[31]
**NiO-NiPi/NF**	1.0 M KOH + 0.50 M Urea	1.349	70.6	[32]
**S-Ni-800**	1.0 M KOH + 0.33 M Urea	1.353	80.9	[33]

Table S3. Comparison on the operation potential of two-electrode systems for UOR.

Catalysts	Electrolyte	Potential (j_{10}, V)	Ref
**Ni_5Nd_1@CC**	1.0 M KOH + 0.33 M Urea	1.398	This work
**O_{vac}-V_1-$Ni(OH)_2$**	1.0 M KOH + 0.33 M Urea	1.50	[1]
**V-$Co_2P_4O_{12}$/CC**	1.0 M KOH + 0.50 M Urea	1.42	[7]
**Ni-MOF-0.5/NF**	1.0 M KOH + 0.50 M Urea	1.52	[9]
**$Ni_{0.864}Co_{0.136}$ LDH**	1.0 M KOH + 0.50 M Urea	1.489	[13]
**CoMn/$CoMn_2O_4$**	1.0 M KOH + 0.50 M Urea	1.51	[14]
**NiFeS@NiMoP/NF**	1.0 M KOH + 0.50 M Urea	1.4	[15]
**V–Ni_3N/N**	1.0 M KOH + 0.50 M Urea	1.42	[16]
**NiS@Ni_3S_2/$NiMoO_4$**	1.0 M KOH + 0.33 M Urea	1.40	[18]
**Ni@NCNT**	1.0 M KOH + 0.50 M Urea	1.56	[19]
**Ni/MNO-10**	1.0 M KOH + 0.50 M Urea	1.45	[22]
**Fe-Ni_3S_2@$FeNi_3$-8**	1.0 M KOH + 0.50 M Urea	1.50	[23]
**Ni_2P/Fe_2P/NF**	1.0 M KOH + 0.50 M Urea	1.47	[24]
**H-NiFe-LDH/NF**	1.0 M KOH + 0.50 M Urea	1.418	[29]
**Ni_3S_2-CDs@NF**	1.0 M KOH + 0.30 M Urea	1.46	[30]
**NiCoCr LDH@NF**	1.0 M KOH + 0.50 M Urea	1.427	[34]
**$Ni(OH)_2$-$NiMoO_x$/NF**	1.0 M KOH + 0.50 M Urea	1.42	[35]
**Ni/NiMoN**	1.0 M KOH + 0.50 M Urea	1.42	[36]

**Mo-Co_3O_4**	1.0 M KOH + 0.30 M Urea	1.42	[37]
**Ru-Ni_3N@NC**	1.0 M KOH + 0.33 M Urea	1.41	[38]
**Mo-NiS**	1.0 M KOH + 0.50 M Urea	1.51	[39]
**Pt-NiS@Ni-CNFs**	1.0 M KOH + 0.33 M Urea	1.44	[40]
**MoS_2/Ni_3S_2**	1.0 M KOH + 0.50 M Urea	1.44	[41]
**NiS/MoS_2@CC**	1.0 M KOH + 0.50 M Urea	1.46	[42]
**NiF_3/Ni_2P@CC-2**	1.0 M KOH + 0.50 M Urea	1.54	[43]
**Ni-S-Se/NF**	1.0 M KOH + 0.50 M Urea	1.47	[44]
**Co_9S_8/Ni_3S_2/NF**	1.0 M KOH + 0.50 M Urea	1.57	[45]
**$NiFeCoS_x$@$FeNi_3$**	1.0 M KOH + 0.33 M Urea	1.42	[46]
**Ni-Mo**	1.0 M KOH + 0.10 M Urea	1.43	[47]
**FeNi Oxide-2**	1.0 M KOH + 0.33 M Urea	1.49	[48]
**Co_3Mo_1S-CC**	1.0 M KOH + 0.50 M Urea	1.50	[49]
**FQD/CoNi-LDH/NF**	1.0 M KOH + 0.50 M Urea	1.45	[50]
**$Ni(OH)_2$-PBA-P**	1.0 M KOH + 0.50 M Urea	1.50	[51]
**$Ni_{1.6}Co_{0.4}P$/C@HCNs/CC**	1.0 M KOH + 0.33 M Urea	1.47	[52]
**Ru-SAC $NiCo_2O_4$**	1.0 M KOH + 0.50 M Urea	1.41	[53]
**Ru@NiON/CNT**	1.0 M KOH + 0.50 M Urea	1.45	[54]
**P-$CoNi_2S_4$**	1.0 M KOH + 0.50 M Urea	1.402	[55]

Table S4. Concentration analysis on the electrolyte after long-term stability test.

Ions	Con. (mg L^{-1})
CO_3^{2-}	0.0608
HCO_3^{2-}	46.6882
NO_2^-	39.9058
NO_3^-	2.9634
OCN^-	0.0103

Reference

[1] H. Qin, Y. Ye, J. Li, W. Jia, S. Zheng, X. Cao, G. Lin, L. Jiao, *Adv. Funct. Mater.* **2023**, *33*, 2209698.

[2] N. N. Rao, C. Alex, S. Tomar, M. S. Naduvil Kovilakath, S. C. Lee, S. Bhattacharjee, N. S. John, *Appl. Catal. B Environ.* **2025**, *371*, 125177.

[3] H. Jiang, J. Xia, L. Jiao, X. Meng, P. Wang, C. S. Lee, W. Zhang, *Appl. Catal. B Environ.* **2022**, *310*, 121352.

[4] M. He, C. Feng, T. Liao, S. Hu, H. Wu, Z. Sun, *ACS Appl. Mater. Interfaces* **2020**, *12*, 2225.

[5] K. Hu, S. Jeong, G. Elumalai, S. Kukunuri, J. I. Fujita, Y. Ito, *ACS Appl. Energy Mater.* **2020**, *3*, 7535.

[6] C. Liu, F. Li, S. Xue, H. Lin, Y. Sun, J. Cao, S. Chen, *ACS Appl. Energy Mater.* **2022**, *5*, 1183.

[7] X. W. Chang, S. Li, L. Wang, L. Dai, Y. P. Wu, X. Q. Wu, Y. Tian, S. Zhang, D. S. Li, *Adv. Funct. Mater.* **2024**, *34*, 2313974.

[8] J. Tang, Z. Li, H. Jang, X. Gu, C. Sun, M. G. Kim, L. Hou, X. Liu, *Adv. Energy Mater.* **2024**, *14*, 2403004.

[9] S. Zheng, Y. Zheng, H. Xue, H. Pang, *Chem. Eng. J.* **2020**, *395*, 125166.

[10] X. Xu, H. Ullah, M. Humayun, L. Li, X. Zhang, M. Bououdina, D. P. Debecker, K. Huo, D. Wang, C. Wang, *Adv. Funct. Mater.* **2023**, *33*, 2303986.

[11] S. Hu, C. Feng, S. Wang, J. Liu, H. Wu, L. Zhang, J. Zhang, *ACS Appl. Mater. Interfaces* **2019**, *11*, 13168.

[12] X. Guo, Y. Li, Z. Xu, D. Liu, A. Kong, R. Liu, *Small* **2025**, *21*, 2408908.

[13] Z. Zheng, D. Wu, L. Chen, S. Chen, H. Wan, G. Chen, N. Zhang, X. Liu, R. Ma, *Appl. Catal. B Environ.* **2024**, *340*, 123214.

[14] C. Wang, H. Lu, Z. Mao, C. Yan, G. Shen, X. Wang, *Adv. Funct. Mater.* **2020**, *30*, 2000556.

[15] S. Yang, T. Wen, Y. Gong, *Int. J. Hydrogen Energy* **2024**, *68*, 834.

[16] R. Q. Li, Q. Liu, Y. Zhou, M. Lu, J. Hou, K. Qu, Y. Zhu, O. Fontaine, *J. Mater. Chem. A* **2021**, *9*, 4159.

[17] M. Sreenivasulu, R. S. Shetti, S. Mathi, T. Maiyalagan, N. P. Shetti, *Mater. Today Sustain.* **2024**, *26*, 100782.

[18] L. Sha, T. Liu, K. Ye, K. Zhu, J. Yan, J. Yin, G. Wang, D. Cao, *J. Mater. Chem. A* **2020**,

8, 18055.

[19] Q. Zhang, F. M. Kazim, S. Ma, K. Qu, M. Li, Y. Wang, H. Hu, W. Cai, Z. Yang, *Appl. Catal. B Environ.* **2021**, *280*, 119436.

[20] N. Wu, X. Zhang, R. Guo, M. Ma, Y. Zhang, T. Hu, *J. Alloys Compd.* **2022**, *903*, 163916.

[21] S. Ni, H. Qu, Z. Xu, X. Zhu, H. Xing, L. Wang, J. Yu, H. Liu, C. Chen, L. Yang, *Appl. Catal. B Environ.* **2021**, *299*, 120638.

[22] V. Maheskumar, A. Min, C. J. Moon, R. A. Senthil, M. Y. Choi, *Small Struct.* **2023**, *4*, 2300212.

[23] W. Zhang, Q. Jia, H. Liang, L. Cui, D. Wei, J. Liu, *Chem. Eng. J.* **2020**, *396*, 125315.

[24] L. Yan, Y. Sun, E. Hu, J. Ning, Y. Zhong, Z. Zhang, Y. Hu, *J. Colloid Interface Sci.* **2019**, *541*, 279.

[25] L. Wu, M. Zhang, Z. Wen, S. Ci, *Chem. Eng. J.* **2020**, *399*, 125728.

[26] G. Qian, J. Chen, L. Luo, H. Zhang, W. Chen, Z. Gao, S. Yin, P. Tsiakaras, *ACS Appl. Mater. Interfaces* **2020**, *12*, 38061.

[27] T. V. M. Sreekanth, B. Naresh, K. Prasad, J. Kim, K. Yoo, *J. Power Sources* **2025**, *653*, 237740.

[28] D. Tavar, S. K. Ojha, Z. Zaidi, H. Prasad, R. K. Sharma, M. Ashiq, M. Mudgal, A. Singh, *ACS Appl. Nano Mater.* **2023**, *6*, 22517.

[29] L. Chen, H. Wang, L. Tan, D. Qiao, X. Liu, Y. Wen, W. Hou, T. Zhan, *J. Colloid Interface Sci.* **2022**, *618*, 141.

[30] Y. Liu, C. Zhou, D. Zhang, J. Wu, A. U. Khan, T. Jiang, M. Liu, H. Sun, H. Liu, B. Mao, *ACS Appl. Nano Mater.* **2024**, *7*, 26145.

[31] Q. Liu, P. Zhao, F. Zhao, J. Zhu, S. Yang, L. Chen, Q. Zhang, *J. Colloid Interface Sci.* **2023**, *644*, 1.

[32] X. Xu, T. Guo, J. Xia, B. Zhao, G. Su, H. Wang, M. Huang, A. Toghan, *Chem. Eng. J.* **2021**, *425*, 130514.

[33] L. Li, L. Wang, X. Peng, S. Tao, M. H. Zeng, *Inorg. Chem. Front.* **2022**, *9*, 1973.

[34] S. Xu, D. Jiao, X. Ruan, Z. Jin, Y. Qiu, Z. Feng, L. Zheng, J. Fan, W. Zheng, X. Cui, *Adv. Funct. Mater.* **2024**, *34*, 2401265.

[35] Z. Dong, F. Lin, Y. Yao, L. Jiao, *Adv. Energy Mater.* **2019**, *9*, 1902703.

[36] Y. Fan, Y. Gu, D. Wang, Y. Jiao, A. Wu, C. Tian, *J. Energy Chem.* **2024**, *95*, 428.

[37] P. Mannu, R. K. Dharman, T. T. T. Nga, A. Mariappan, Y. C. Shao, H. Ishii, Y. C. Huang, A. Kandasami, T. H. Oh, W. C. Chou, C. L. Chen, J. L. Chen, C. L. Dong, *Small*

2024, *21*, 2403744.
[38] Y. Liu, D. Zheng, Y. Zhao, P. Shen, Y. Du, W. Xiao, Y. Du, Y. Fu, Z. Wu, L. Wang, *Int. J. Hydrogen Energy* **2022**, *47*, 25081.
[39] Y. Zhou, Y. Wang, D. Kong, Q. Zhao, L. Zhao, J. Zhang, X. Chen, Y. Li, Y. Xu, C. Meng, *Adv. Funct. Mater.* **2023**, *33*, 2210656.
[40] M. Zhong, J. Yang, M. Xu, S. Ren, X. Chen, C. Wang, M. Gao, X. Lu, *Small* **2024**, *20*, 2304782.
[41] Y. Ren, C. Wang, W. Duan, L. Zhou, X. Pang, D. Wang, Y. Zhen, C. Yang, Z. Gao, *J. Colloid Interface Sci.* **2022**, *628*, 446.
[42] C. Gu, G. Zhou, J. Yang, H. Pang, M. Zhang, Q. Zhao, X. Gu, S. Tian, J. Zhang, L. Xu, Y. Tang, *Chem. Eng. J.* **2022**, *443*, 136321.
[43] K. Wang, W. Huang, Q. Cao, Y. Zhao, X. Sun, R. Ding, W. Lin, E. Liu, P. Gao, *Chem. Eng. J.* **2021**, *427*, 130865.
[44] N. Chen, Y. X. Du, G. Zhang, W. T. Lu, F. F. Cao, *Nano Energy* **2021**, *81*, 105605.
[45] Y. Bao, Y. Yang, J. Jiang, J. Gao, Y. He, S. Wang, X. Bai, X. Dong, H. Tao, *ACS Appl. Nano Mater.* **2025**, *8*, 6530.
[46] J. Shen, Q. Li, W. Zhang, Z. Cai, L. Cui, X. Liu, J. Liu, J. Liu, X. Liu, *J. Mater. Chem. A* **2022**, *10*, 5442.
[47] J. Y. Zhang, T. He, M. Wang, R. Qi, Y. Yan, Z. Dong, H. Liu, H. Wang, B. Y. Xia, *Nano Energy* **2019**, *60*, 894.
[48] X. Gu, D. Yang, Z. Liu, S. Wang, L. Feng, *Electrochim. Acta* **2020**, *353*, 136516.
[49] P. Li, Z. Zhuang, C. Du, D. Xiang, F. Zheng, Z. Zhang, Z. Fang, J. Guo, S. Zhu, W. Chen, *ACS Appl. Mater. Interfaces* **2020**, *12*, 40194.
[50] Y. Feng, X. Wang, J. Huang, P. Dong, J. Ji, J. Li, L. Cao, L. Feng, P. Jin, C. Wang, *Chem. Eng. J.* **2020**, *390*, 124525.
[51] H. Xu, K. Ye, K. Zhu, Y. Gao, J. Yin, J. Yan, G. Wang, D. Cao, *ACS Sustain. Chem. Eng.* **2020**, *8*, 16037.
[52] S. Rezaee, S. Shahrokhian, *Nanoscale* **2020**, *12*, 16123.
[53] A. Gupta, S. Ghosh, D. Bhalothia, S. Thangarasu, B. Ghosh, R. Urkude, J. Chowdhury, S. Pande, *J. Mater. Chem. A* **2024**, *12*, 23819.
[54] H. J. Kim, R. Santhosh Kumar, S. Tamilarasi, S. Vijayapradeep, H. Bin Kwak, D. Jin Yoo, *Chem. Eng. J.* **2024**, *489*, 151003.
[55] X. F. Lu, S. L. Zhang, W. L. Sim, S. Gao, X. W. Lou, *Angew. Chem. Int. Ed.* **2021**, *60*, 22885.
